# Optimization and Control of Large Block Copolymer Self-Assembly via Precision Solvent Vapor Annealing

**DOI:** 10.1021/acs.macromol.0c02543

**Published:** 2021-01-22

**Authors:** Andrew Selkirk, Nadezda Prochukhan, Ross Lundy, Cian Cummins, Riley Gatensby, Rachel Kilbride, Andrew Parnell, Jhonattan Baez Vasquez, Michael Morris, Parvaneh Mokarian-Tabari

**Affiliations:** † Advanced Material and BioEngineering Research Centre (AMBER), Trinity College Dublin, The University of Dublin, Dublin 2, Ireland; ‡ School of Chemistry, Trinity College Dublin, The University of Dublin, Dublin 2, Ireland; § CNRS, Bordeaux INP, LCPO, UMR 5629 and CNRS, Centre de Recherche Paul Pascal, UMR 5031, Université de Bordeaux, Pessac F-33600, France; ∥ Department of Physics and Astronomy, University of Sheffield, Sheffield S3 7RH, U.K.

## Abstract

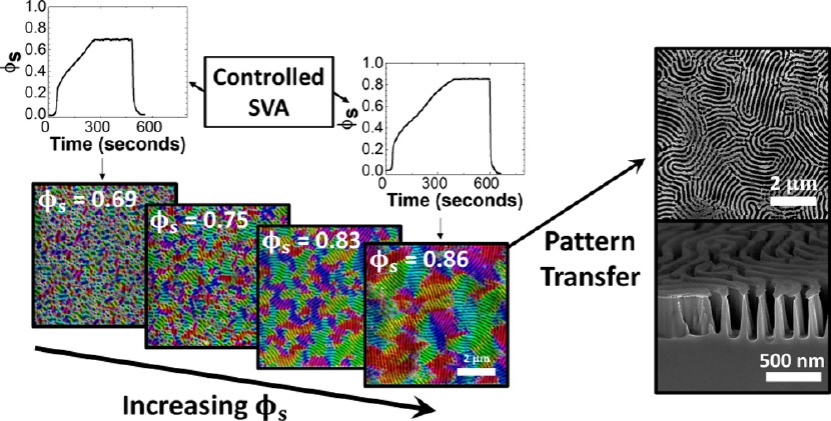

The self-assembly of ultra-high molecular weight (UHMW) block copolymers (BCPs) remains a complex and time-consuming endeavor owing to the high kinetic penalties associated with long polymer chain entanglement. In this work, we report a unique strategy of overcoming these kinetic barriers through precision solvent annealing of an UHMW polystyrene-*block*-poly(2-vinylpyridine) BCP system (*M*_w_: ∼800 kg/mol) by fast swelling to very high levels of solvent concentration (ϕ_s_). Phase separation on timescales of ∼10 min is demonstrated once a thickness-dependent threshold ϕ_s_ value of ∼0.80–0.86 is achieved, resulting in lamellar feature spacings of over 190 nm. The threshold ϕ_s_ value was found to be greater for films with higher dry thickness (*D*_0_) values. Tunability of the domain morphology is achieved through controlled variation of both *D*_0_ and ϕ_s_, with the kinetically unstable hexagonal perforated lamellar (HPL) phase observed at ϕ_s_ values of ∼0.67 and *D*_0_ values of 59–110 nm. This HPL phase can be controllably induced into an order–order transition to a lamellar morphology upon further increase of ϕ_s_ to 0.80 or above. As confirmed by grazing-incidence small-angle X-ray scattering, the lateral ordering of the lamellar domains is shown to improve with increasing ϕ_s_ up to a maximum value at which the films transition to a disordered state. Thicker films are shown to possess a higher maximum ϕ_s_ value before transitioning to a disordered state. The swelling rate is shown to moderately influence the lateral ordering of the phase-separated structures, while the amount of hold time at a particular value of ϕ_s_ does not notably enhance the phase separation process. These large period self-assembled lamellar domains are then employed to facilitate pattern transfer using a liquid-phase infiltration method, followed by plasma etching, generating ordered, high aspect ratio Si nanowall structures with spacings of ∼190 nm and heights of up to ∼500 nm. This work underpins the feasibility of a room-temperature, solvent-based annealing approach for the reliable and scalable fabrication of sub-wavelength nanostructures via BCP lithography.

## Introduction

The directed self-assembly of block copolymers (BCPs) is a well-studied technique for the controlled formation of a wide range of periodic thin-film morphologies, including spherical, cylindrical, gyroidal, and lamellar, among others.^1^ Structural modulation can be achieved through the manipulation of the molecular composition, while the periodicity of the domains can be adjusted through varying the molecular weight.^2,3^ From a technological standpoint, the high degree of versatility of BCP self-assembly, in addition to scalability and low process temperatures, has provided an attractive route for the cost-effective fabrication of surface nanostructures with an enormous variety of potential applications, including nanoelectronics,^4,5^ chemical sensors,^6−9^ antireflective coatings,^10−13^ and optically active surfaces.^14−16^ In the case of optoelectronic applications such as interconnect patterning, the periodicity of the lateral domain features must generally exceed approximately 100 nm such that the structures are capable of interacting with wavelengths on the order of visible light.^17,18^ In order to facilitate the formation of such feature sizes, ultra-high molecular weight (UHMW) BCP systems in excess of 500 kg/mol are typically utilized. The self-assembly of UHMW BCP systems creates additional complexities in the annealing process, in particular the extremely slow ordering kinetics associated with increased chain entanglement.^19^ The energy barrier required to induce chain mobility in highly entangled BCPs cannot always be overcome even at temperatures exceeding that of the glass transition temperature (*T*_g_) of the BCP system, effectively eliminating the possibility of a purely thermal annealing approach in such UHMW systems.^20^

The majority of recent literature, including the work described here, instead employs solvent vapor annealing (SVA) as an alternative technique to facilitate the self-assembly of UHMW systems. SVA involves the uptake of solvent into a BCP film, resulting in increased polymer chain mobility, a lower effective value of *T*_g_, and the avoidance of any thermal degradation of the material.^21−23^ There are multiple interdependent variables that influence the SVA process, including pressure, temperature, and humidity. Nonetheless, previous work has shown that it can be performed with a very simple strategy known as “static” SVA—this consists of a BCP sample placed inside a sealed chamber containing a reservoir of solvent, which is then left for a predetermined period of time.^24−27^ Moreover, SVA can be performed at room temperatures or below and has been proven to effectively induce self-assembly in a number of UHMW BCP systems.^20,28−31^ Kim et al. successfully obtained phase separation of lamellar and gyroid UHMW polystyrene-*block*-poly(methyl methacrylate) (PS-*b*-PMMA) BCP films with periods of ∼200 nm using tetrahydrofuran (THF) as a neutral solvent for SVA, followed by a 12 h thermal annealing step.^20,29^ Phase separation of a UHMW spherical PS-*b*-PMMA system was also demonstrated by Cao et al. again using THF as the annealing solvent of choice.^28^ Additionally, Takano et al. developed a novel instrumentation technique to monitor the phase separation of UHMW lamellar PS-*b*-PMMA using in situ atomic force microscopy (AFM) under high swelling conditions during SVA.^30^ Most recently, Cummins et al. achieved the phase separation of a high molecular weight PS-P2VP system (*M*_n_ = 430 kg/mol) in a time period of 1 h using an uncontrolled “static” SVA strategy.^32^ Despite the relative success of these previous studies for the induction of phase separation in UHMW systems, the required timescales often extended from several hours to days—thereby severely hindering the industrial applicability of such processes.^20,28−30^ The acceleration of the SVA process is therefore critical for the future development of this field. Here, we demonstrate a procedure for ultrafast self-assembly of UHMW BCP systems by controlling the swelling kinetics during SVA, reducing the required annealing time to minutes.

One notable improvement in expediting the SVA process for UHMW systems as of late was by Doerk et al., who utilized blends consisting of lamella-forming PS-*b*-PMMA (*M*_n_ ≤ 2000 kg/mol) combined with low molecular weight PS and PMMA homopolymer to accelerate the phase separation process.^31^ This resulted in the formation of ordered lamellar domains with a periodicity of up to 211 nm from a total annealing time of 1 h (plus an additional 5 min thermal annealing step following SVA). Although this work demonstrated a major step toward accelerated SVA time frames for UHMW BCPs, the influence of many of the kinetic components of the SVA technique—such as the swelling/deswelling rate or swelling time—remains predominantly unexplored for such systems. Previous kinetic studies examining lower molecular weight BCPs have proven the critical importance of the aforementioned parameters in the enhancement of self-assembly timescales;^33−37^ hence, it seemed desirable to determine the effects of such variables on an UHMW system.

In order to maintain precise control over BCP swelling kinetics during SVA, a variety of specialized annealing chambers have been constructed where the solvent uptake into the BCP film can be regulated in situ during annealing.^33,35,38−42^ The primary kinetic variable of focus for many of these setups is the swollen film thickness *D*_sw_, which is dependent on the concentration of the solvent inside the polymer film ϕ_s_. *D*_sw_ is well known to play a crucial role in the kinetics of self-assembly, with a positive observed correlation between a higher value of *D*_sw_ and increased lateral ordering.^35,37,43^ Recently, Hulkkonen et al. utilized a custom-built annealing chamber to precisely control the swelling behavior of HMW PS-*b*-P2VP systems (*M*_n_ = 258 kg/mol) via a temperature-controlled sample stage, resulting in the formation of hexagonal cylindrical domains in <15 minutes.^35^ The BCP systems examined in their study, however, were not of sufficient molecular weight to fabricate domain sizes greater than 100 nm. To our knowledge, little published work exists on optimizing the self-assembly kinetics of UHMW BCP systems using controlled SVA techniques.

Accordingly, in this report, we investigate the swelling kinetics of a commercially available UHMW lamellar PS-*b*-P2VP BCP system with a molecular weight of ∼800 kg/mol (*M*_n_: 440–353 kg/mol, *f*_PS_ = 0.57) in order to improve the reliability and speed of the microphase separation process. This particular BCP system was chosen as previous published work by our group demonstrated the potential self-assembly of this system upon exposure to both THF and chloroform using the conventional “static” SVA method.^12^ In the work shown herein, we demonstrate a greatly expedited timescale for the phase separation of UHMW PS-*b*-P2VP films using a bespoke SVA chamber. We first examine the effects of the dry film thickness, *D*_0_, and the solvent concentration in the polymer film, ϕ_s_, on the structural evolution and lateral grain ordering of the rapidly swollen BCP films, resulting in the controlled formation of both equilibrium and non-equilibrium BCP phases. The formation of the previously demonstrated hexagonal perforated lamellar (HPL) phase along with well-ordered lamellar domains with periods of ∼190 nm is achieved using total annealing times of ∼10 min. The effect of swelling time on the structural evolution, along with the influence of the swelling rate on the lateral grain sizes of the lamellar domains, is also examined. Finally, we also show the capability of the phase-separated films to be utilized for pattern transfer, with the formation of high aspect ratio (up to 7.5) sub-wavelength Si nanostructures using a metal salt infiltration process, followed by reactive ion etching. This is a critical asset for application areas.

## Results and Discussion

A 793 kg/mol PS-*b*-P2VP (440 kg/mol PS, 353 kg/mol P2VP) system was utilized for this study. This system was of particular interest as it was shown in previous work by our group to be capable of phase separation into a kinetically unstable HPL structure upon “static” SVA in a THF and chloroform atmosphere for an hour, with a feature spacing of ∼180 nm.^12^ This structure, with large area coverage and sub-wavelength periodicity, proved to be highly applicable in the fabrication of antireflective nanostructures. One issue that was encountered, however, was the low reproducibility of the self-assembly method. It was found that slight fluctuations in the lab temperature, annealing solvent concentration, or even the position of the sample within the annealing chamber often led to widely different morphologies. Although the variation in results was expected due to the non-equilibrium nature of microphase separation UHMW BCPs, it was of interest from a technological point of view to examine and monitor the self-assembly kinetics in more detail in order to achieve greater repeatability.

UHMW BCP systems typically require high swelling ratios in order to initiate polymer mobility.^44^ This is due to the influence of molecular weight on the level of chain entanglement—higher molecular weight systems possess longer polymer chains and thereby a higher degree of entanglement in the dry BCP film. SVA is a well-known method that can be used to address this issue. With the addition of a relatively neutral solvent to the BCP film via SVA, the polymer–polymer interactions can be reduced as the solvent molecules produce a screening effect at the interface between the two blocks.^45^ This screening effect can be quantitatively represented by an effective interaction parameter χ_eff_^46^
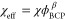
1where χ is the Flory–Huggins interaction parameter for the dry BCP film, ϕ_BCP_ is the polymer concentration of the swollen film, and β is an exponent factor with a value that varies between ∼1 and ∼2 and varies depending on the morphology of the ordered film, the selectivity of the solvent, and the solvent concentration inside the film.^47,48^ A higher swelling ratio (and consequently a lower value of ϕ_BCP_) will therefore reduce the value of χ_eff_, increasing chain mobility and allowing microphase separation to occur. ϕ_BCP_ is equal to (*D*_0_/*D*_sw_), the inverse of the swelling ratio, where *D*_0_ is the initial film thickness, and *D*_sw_ is the swollen film thickness. This value can also be related to the solvent concentration inside the BCP film, ϕ_s_, which is equal to 1 – ϕ_BCP_.^39^

**Figure 1:**
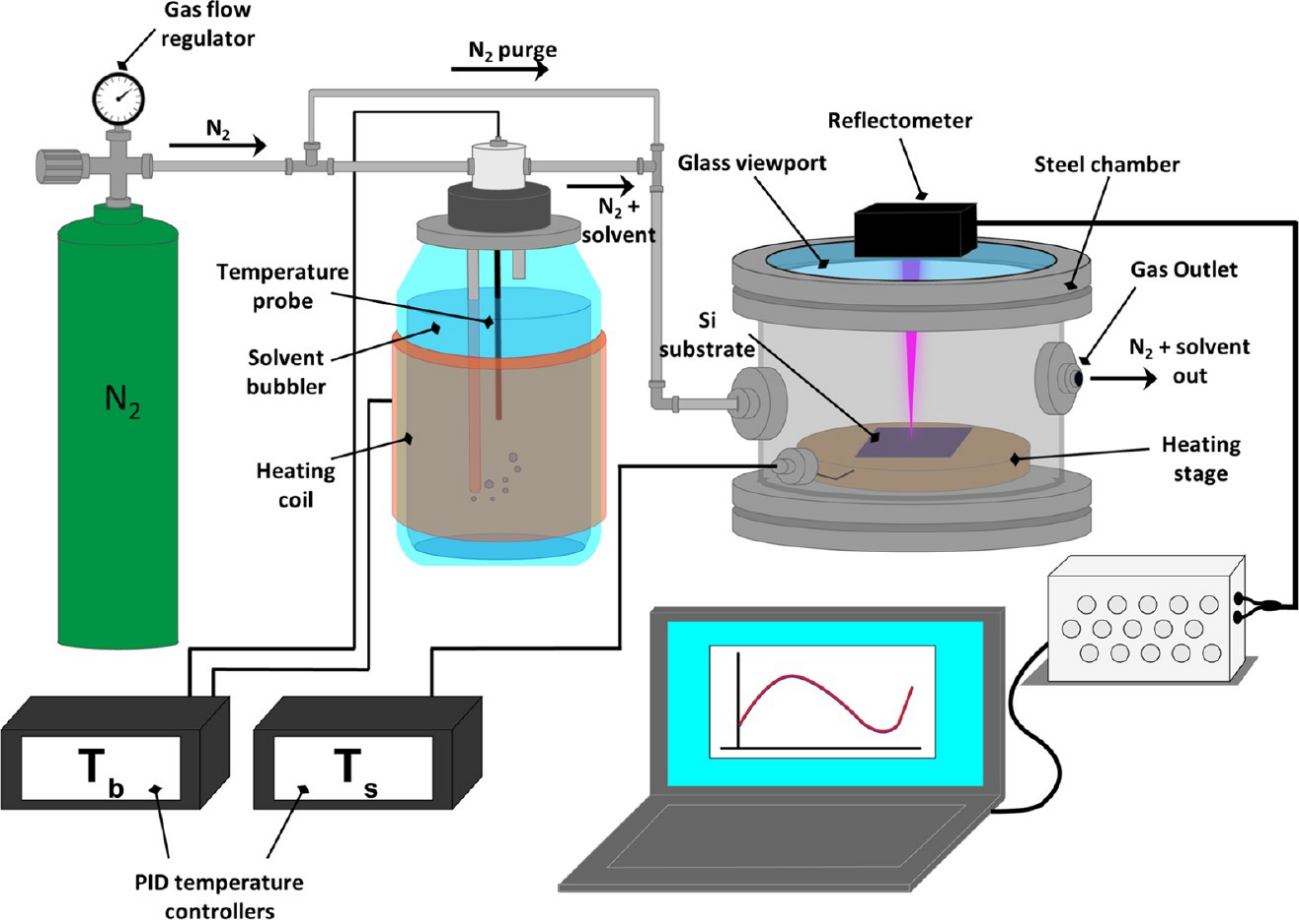
Diagram of the SVA setup for UHMW films. Films of PS-*b*-P2VP are spin-coated onto Si substrates and then solvent vapor annealed inside the chamber while the film thickness is monitored in situ using a reflectometer.

Conventional SVA methods are often incapable of attaining the minimum swelling ratio required to initiate phase separation of UHMW systems regardless of the total annealing time due to a lack of control over the relative saturation *P*/*P*_sat_ of the solvent vapor in the chamber (where *P* is the partial pressure of the annealing solvent vapor and *P*_sat_ is the saturated vapor pressure of the solvent at a fixed temperature).^35^ Control over the relative saturation of a BCP film during SVA is crucial in order to attain high swelling ratios, as *P*/*P*_sat_ is directly related to ϕ_s_ through the following relationship^49^
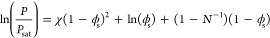
2where *N* is the degree of polymerization. Consequently, in order to fully examine the swelling kinetics of the UHMW BCP system, we utilized a customized SVA rig system where *P*/*P*_sat_ of the annealing chamber (and thereby the solvent uptake ϕ_s_ at any given time) could be carefully monitored in situ by varying the temperature of the sample stage (±0.1 °C) as shown schematically in Figure 1. By incrementally decreasing the temperature of the stage, the *P*/*P*_sat_ in the localized area of the BCP film is increased, leading to a greater degree of solvent uptake. Conversely, if the stage temperature is increased, the *P*/*P*_sat_ will decrease inducing solvent expulsion from the film.^35,38^ This setup is an upgraded version of the system described by Lundy et al., with the addition of a reflectometer that allows in situ monitoring of film swelling.^38^

Various kinetic parameters of the film swelling experiments such as the swelling ratio, swelling time, and rate of swelling were controlled through precise variation of the stage temperature (±0.1 °C) while maintaining a constant solvent temperature of 21.0 ± 0.1 °C in the bubbler. The noted degree of accuracy for the stage temperature is essential for the controlled swelling of BCP films to high values of ϕ_s_, as even small fluctuations (>0.25 °C) in sample temperature at high swelling regimes can result in dramatic variations in the swollen film thickness.^35^ The setup was contained in a fume hood that was maintained at a lab temperature of ∼21 ± 1 °C. By decreasing the temperature of the stage, the relative saturation of the solvent vapor was increased, thus increasing the swelling ratio of the film.^23^

The annealing solvent of choice for this study was a blend of THF and chloroform, both of which are relatively non-selective to both PS and P2VP segments (slight selectivity of THF to PS and chloroform to P2VP).^37,49^ A range of solvent blend ratios were trialed in order to optimize the phase separation of the BCP system (see Section S1), of which a 2:1 molar ratio of chloroform to THF was chosen as the optimal blend ratio for the kinetic studies described below. This was calculated to give a ratio of approximately 82:18 of chloroform to THF in the vapor phase due to the non-ideality of the solvent mixture.^50^ Both THF and chloroform have high vapor pressures with values that are relatively close at 21 °C (136 and 165 mmHg, respectively, see Section S1); therefore, it was assumed that the mole fractions of both solvents within the bubbler did not vary significantly over the annealing timescales analyzed in this work. A full description of the solvent variation experiments, including AFM images of samples along with a calculation of the resulting vapor-phase mole fractions accounting for the nonideality of the mixture, is contained in Section S1. A detailed analysis of the effect of solvent mixtures will be the subject of a future study.

**Figure 2:**
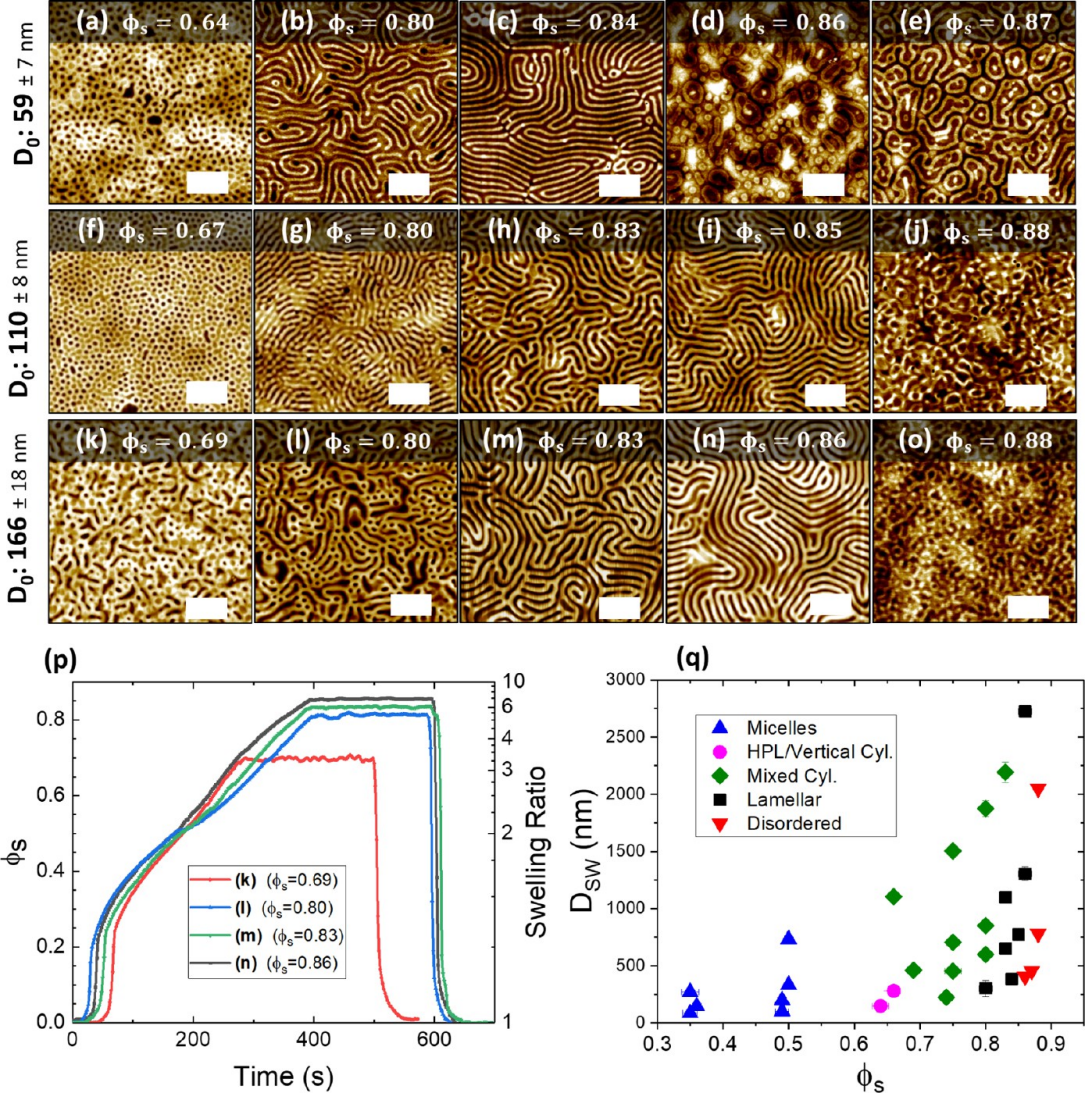
AFM images (a–o) show PS-*b*-P2VP films of varying thicknesses annealed in 2:1 chloroform/THF vapor up to a range of solvent concentration (ϕ_s_) values. All scale bars are 1 μm. Once the targeted value of ϕ_s_ was reached, the films were held at this value for approximately 200 s before being rapidly quenched in order to preserve the phase-separated structure. (p) Examples of solvent uptake plots for (k–n) during SVA, with the corresponding ϕ_s_ value shown in the inset that the film was held at for 200 s. (q) is an orientation diagram of the swollen film thickness *D*_sw_ as a function of ϕ_s_, with the color of each data point conveying the resulting morphology of the film post-SVA.

As a starting point for this kinetic study, we decided to examine the effects of varying both *D*_0_ and ϕ_S_ on the resulting phase-separated morphologies of the BCP system. Figure 2a–o shows a set of AFM images of the PS-*b*-P2VP BCP film annealed as a function of both *D*_0_ and ϕ_s_. The value of *D*_0_ was varied by adjusting the concentration of the BCP dissolved in solution prior to spin coating (from 1 to 3 w/w %), giving an initial thickness range of ∼59 to 371 nm. The films were swollen to a range of ϕ_S_ values, calculated from in situ monitoring of film thickness during SVA, and held at the set value for approximately 200 s before rapid deswelling through N_2_ purging of the chamber. Rapid quenching of the solvent is essential to “kinetically trap” the film morphology in the swollen state upon drying.^35,51,52^ The temperature conditions for the films were kept as constant as possible to ensure that the swelling rate remained similar for all films, with an initial stage temperature of 19.9 °C and a bubbler temperature of 21.0 °C. Some slight variation in swelling profiles between samples is noted (Figure 2p), which we attribute to minor daily temperature fluctuations in the lab environment. To avoid any excessive fluctuation in ϕ_s_ during the 200 s hold time, the stage temperature was manually varied in increments of ±0.1 °C. The total annealing times were between ∼8 and 11 min, with samples held at higher ϕ_s_ values requiring slightly longer swelling times as shown in the example set of in situ swelling plots for the 166 nm films (Figure 2p).

A significant evolution of the film morphology was observed as the value of ϕ_s_ (that the films were held at) was increased. Below a ϕ_s_ value of ∼0.64, no structural change was observed for any value of *D*_0_, with the films remaining in an apparent vitrified micellar state as seen in previous studies.^39,43^ At ϕ_s_ values between ∼0.64 and 0.67, as can be seen in Figure 2a,e, a perpendicular cylindrical surface structure emerged for *D*_0_ values of ∼59 and 118 nm. We suggest that this is a HPL structure, as we previously observed through static SVA of this system.^12^ The formation of the HPL phase is attributed to confinement effects as the film thickness decreases below the domain period *L*_0_ and the limited maneuverability of the polymer chains at low values of ϕ_s_.^27,53^ As the thickness of the unswollen dry BCP film moves toward commensurability (*D*_0_ ≥ ∼166 nm), as was the case in Figure 2k, the HPL phase was not observed at the same ϕ_s_ values and a poorly ordered mixed cylindrical structure was instead noted. As ϕ_s_ was further increased to between ∼0.80 and 0.87, well-developed lamellar structures were observed to emerge at all *D*_0_ values. The domain period *L*_0_ of these structures was determined to be 193 nm from power spectrum density (PSD) plots of AFM images (see Figure S7). Interestingly, we observed a slight correlation between the critical ϕ_s_ value at which the onset of phase separation occurred (ϕ_s,c_) and *D*_0_, with the onset shifting toward lower ϕ_s_ values for thinner films. This is likely due to a reduction in the amount of entangled material required to rearrange at lower *D*_0_ values, which has been shown in previous work to result in faster ordering kinetics.^54^ We attribute the high observed value of ϕ_s,c_ to the larger chain lengths and subsequent high level of entanglement associated with this UHMW system.

In the case of the films with *D*_0_ = 59–118 nm, the HPL phase (Figure 2a,e) was observed to undergo an order–order transition to a lamellar structure (Figure 2b,h) upon increasing ϕ_s_, in agreement with previous work.^55^ The ordering of the lamellar domains improved with increasing ϕ_s_ up to a *D*_0_-dependent value of ∼0.84 to 0.87. Upon further swelling above these values, the films appeared to undergo an order–disorder transition (ODT) with the loss of any observable surface ordering. Additionally, maintaining regular ϕ_s_ values above 0.87 was found to be difficult for all film thicknesses due to the intermittent breakdown of the reflectometer model, which we attribute to partial dewetting of the films during SVA. A slight thickness dependence was observed for the value of ϕ_s_ at the ODT (henceforth referred to as ϕ_s,ODT_), with lower *D*_0_ resulting in a slightly lower ϕ_s,ODT_. A likely explanation for this dependency is the reduction in polymeric material, resulting in less required molecular rearrangement, as mentioned previously.

**Figure 3:**
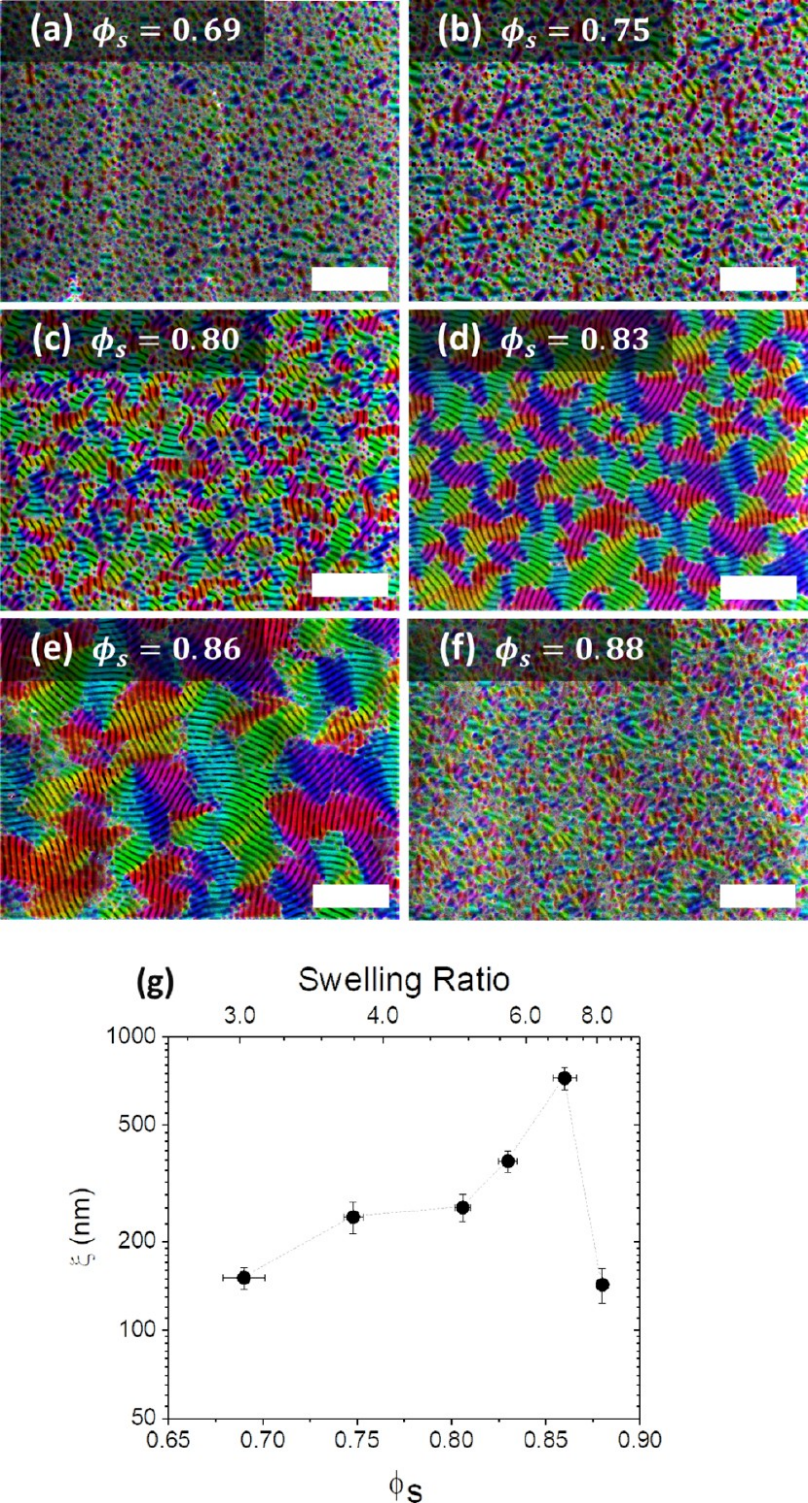
(a–d) Orientational mapping of scanning electron microscopy (SEM) images showing grains parallel to the surface after swelling to different values of solvent concentration (ϕ_s_): (a) ϕ_s_ = 0.69, (b) ϕ_s_ = 0.75, (c) ϕ_s_ = 0.80, (d) ϕ_s_ = 0.83, (e) ϕ_s_ = 0.86, (f) ϕ_s_ = 0.88, and (g) correlation length ξ as a function of ϕ_s_ and swelling ratio. Scale bars are 2 μm.

The complete range of ϕ_s_ values and their resulting morphologies are summarized in the orientation diagram shown in Figure 2n, with their corresponding AFM images available in Figure S8. The effect of ϕ_s_ on the lateral ordering is more closely examined in Figure 3.

In order to quantify the effect of ϕ_s_ on the lateral ordering of the lamellar features, we used image analysis software to generate orientation maps of the BCP films after SVA. Figure 3a–f shows colorized SEM images of the PS-*b*-P2VP films with *D*_0_ = ∼166 nm swollen to and held at various values of ϕ_s_ (0.69–0.88) for 200 s, followed by rapid deswelling (same annealing conditions as in Figure 2), with the coloring representing the orientation of the lamellar microdomains. The orientational ordering of the films increases with the ϕ_s_ value reached during swelling, with significant microdomain orientation only observed at ϕ_s_ = ∼0.83 or higher. The maximum grain size was achieved at ϕ_s_ = ∼0.86, with complete disordering of the phase-separated lamellar structure observed at ϕ_s_ = ∼0.88. The ODT value of ϕ_s_ can therefore be cautiously estimated to lie close to the value of ϕ_s_ = ∼0.87. The grain size was further quantitatively characterized by determining the microdomain correlation length ξ for the lateral ordering of the lamellar structures for each sample, as shown in Figure 3g.^54,56^ At low levels of ϕ_s_, the value of ξ remains at a value close to the domain period of ∼190 nm. The highest value of ξ = 723 ± 62 nm was obtained through annealing at the closest possible ϕ_s_ value (0.86) to our estimated ODT concentration of ϕ_s_ = ∼0.87. Once the swelling surpassed this point, as was the case with Figure 3f where ϕ_s_ = 0.88, long-range ordering was lost and the value of ξ returned to the order of roughly a single domain period.

**Figure 4:**
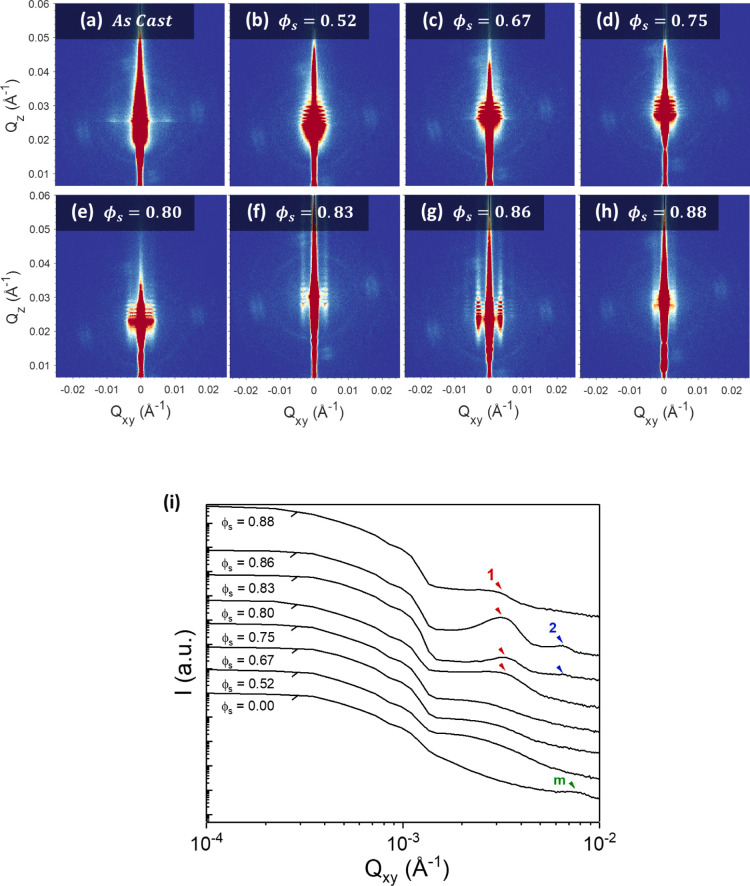
(a–h) 2-dimensional GISAXS scattering patterns showing the morphological evolution of the 166 nm PS-*b*-P2VP films swollen to different values of ϕ_S_. Films were held at the noted value of ϕ_s_ for ∼200 s before rapid deswelling. (i) 1D Intensity profiles extracted from the GISAXS images at the determined Yoneda position for each sample. First-order, second-order scattering peaks are marked as 1 and 2, and scattering peak for as-cast micelle film as *m*.

Grazing-incidence small-angle X-ray scattering (GISAXS) was then used to examine the effect of ϕ_s_ on the internal structure of the 166 nm BCP films over macroscopic areas. Measurements were taken of the films after rapid deswelling with no subsequent processing, as shown in the GISAXS images in Figure 4a–h along with the 1D in-plane intensity profiles in Figure 4i. The 1D intensity profiles were extracted from the GISAXS images at the determined Yoneda position for each sample. A clear evolution of morphology can be observed as ϕ_s_ increases. For the as-cast film, a very weak scattering pattern is evident in Figure 4a—this indicates a mainly disordered film, which can be attributed to the kinetically trapped state after rapid solvent evaporation during spin coating.^57^ A weak scattering peak is evident in Figure 4i (marked as *m* in green) at a *Q*_*xy*_ value of ∼7.98 × 10^–3^ Å^–1^, which corresponds to a domain spacing *d* of ∼79 nm (using *d* = 2π/*Q*_*xy*_); this likely originates from the micellar structures evident in AFM images of the as-cast film surface (shown in Figure S7). For films swollen to a ϕ_s_ value of between 0.52 and 0.75 (Figure 4b–d), no notable scattering peaks are observed, which suggests that the BCP chains are still too entangled at this level of swelling to self-assemble into well-defined microdomains. Upon reaching a ϕ_s_ value of 0.80 (Figure 4e), a first-order scattering peak (marked as 1 in red) begins to emerge at a *Q*_*xy*_ value of approximately 3.33 × 10^–3^ Å^–1^. This peak sharpens and intensifies in strength along *Q*_*z*_ as ϕ_s_ is increased to 0.83 and then to 0.86 (Figure 4f,g), indicating a structural transition to a perpendicular lamellar morphology once the films exceed the threshold ϕ_s_ value of ∼0.8. The extension of the perpendicular lamellar morphology throughout the entire thickness of the film is further proven by cross-sectional FIB/SEM analysis (see Figure S10). The in-plane domain spacing *d* at ϕ_s_ = 0.83 is calculated to be approximately ∼184 nm (*Q*_*xy*_ = 3.41 × 10^–3^ Å^–1^), while at ϕ_s_ = 0.86, this increases to 191 nm (*Q*_*xy*_ = 3.29 × 10^–3^ Å^–1^). These values are in close agreement with lamellar spacing values calculated from AFM (see Figure S8). A weak second-order peak is also visible at 2*Q*_xy_^*^ (marked as 2 in blue in Figure 4i), which again is indicative of the development of well-ordered perpendicular lamellar domains in the film.^58^ Once the value of ϕ_s_ is increased to 0.88 (Figure 4h), the first-order peak is observed to diminish and the second-order peak vanishes. This infers a loss of structural ordering throughout the BCP film as it undergoes an ODT, which is in agreement with the surface transition shown in AFM images (see Figure 2n,o). Oscillations in *Q*_*z*_ can be observed for all ϕ_S_ values between 0.52 and 0.86, which we suggest arise from surface roughness correlation effects between the polymer film and the substrate.^59^

**Figure 5:**
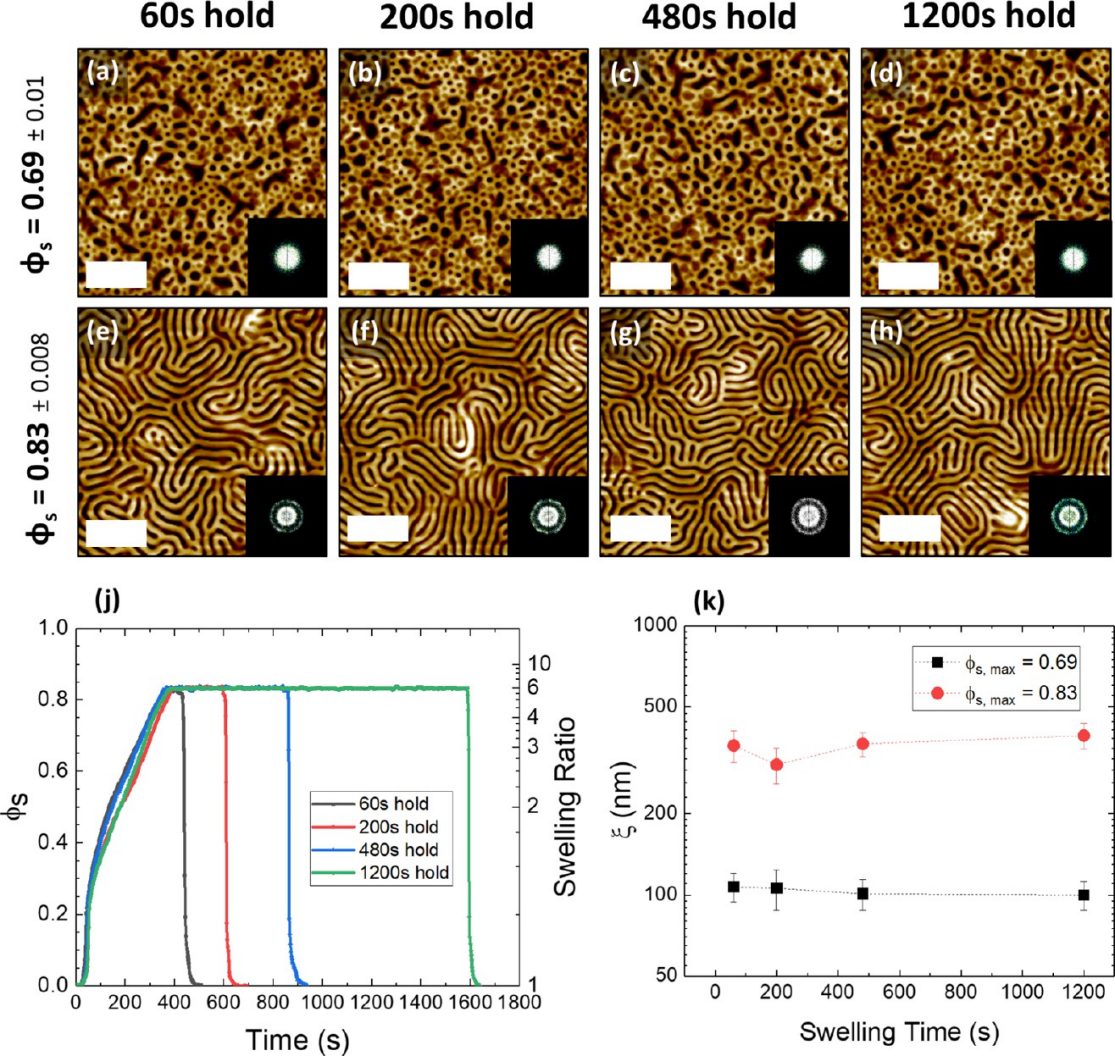
AFM images (a–d) show PS-*b*-P2VP films annealed to a solvent concentration (ϕ_s_) value of 0.69 and held at this value for different amounts of time (ST = 60, 200, 480, and 1200 s). (e–h) are annealed to ϕ_s_ = 0.83 and held for the same timescales. Corresponding Fourier transforms are shown in the inset. (j) The swelling profiles for (e–h), as a function of ϕ_s_ and swelling ratio. (k) The variation of the correlation length ξ with swelling time. All scale bars are 1 μm.

In order to fully optimize and understand the annealing process, it was critical to determine the effect of annealing time on the relative ordering and morphologies of the BCP films. A series of BCP films with a *D*_0_ value of ∼166 nm were swollen to either ∼3*D*_0_ (ϕ_s_ = 0.69) or ∼6*D*_0_ (ϕ_S_ = 0.83) and held at that swelling ratio for varying amounts of time between 60 and 1200 s, as shown in Figure 5j for the ϕ_S_ = 0.83 samples (ϕ_s_ = 0.69 profiles shown in Figure S4). The rate of swelling was maintained by controlling the stage and bubbler temperatures. For both values of ϕ_s_, it was observed that the amount of time the BCP was held at a particular solvent concentration did not significantly affect the morphology or ordering of the resulting structure. In the case of Figure 5a–d, where the films were swollen to 3*D*_0_, the increased swelling time did not result in a more ordered or developed structure. This is furthermore confirmed by the low level of variation in the value of ξ in Figure 5k. In the case of films swollen to 6*D*_0_, phase separation was induced, but again, the increase in swelling time did not result in any observed increase in lateral ordering, and the value of ξ remained with the range of approximately 300–400 nm. These results reinforce the existence of a minimum ϕ_s_ value, below which the BCP films will remain in a vitrified state regardless of the annealing time.^51^ This observation is also in agreement with previous work on lower molecular weight cylinder-forming PS-*b*-P2VP systems that showed minimal change in the ξ with longer annealing times.^43^

**Figure 6:**
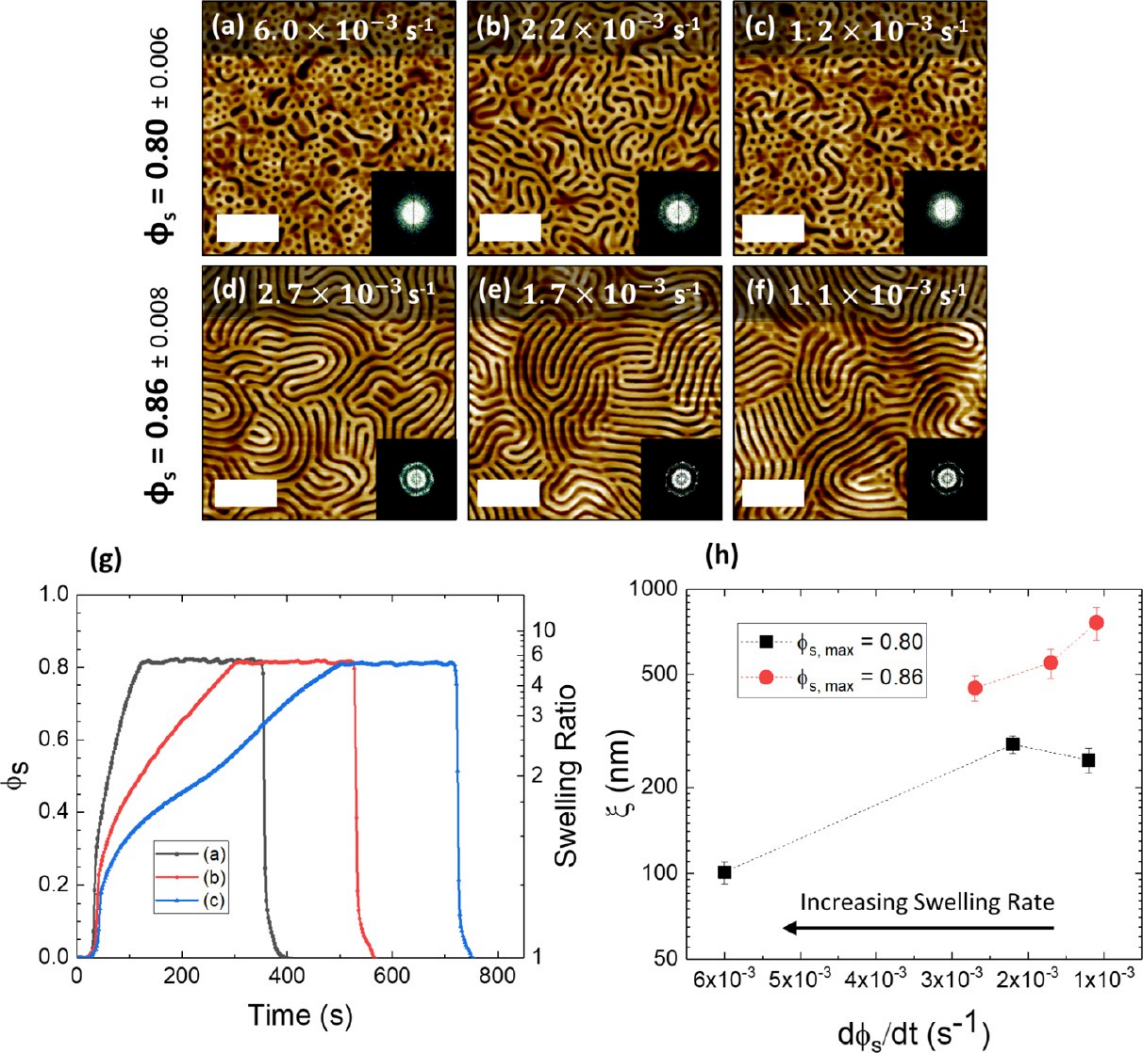
The effect of the rate of solvent uptake (dϕ/d*t*) on lateral ordering for two different values of ϕ_s_. AFM images of films with different swelling rates for film reaching maximum ϕ_s_ = 0.8 (a–c) and maximum ϕ_s_ = 0.86 (d–f). Fourier transforms are shown in the inset. (g) Solvent annealing profile for (a–c) as a function of ϕ_s_ and swelling ratio at different swelling rates. (h) Correlation length ξ at different swelling rates, indicating that the slower swelling rate improves the microdomain correlation length. All scale bars are 1 μm.

The influence of the swelling kinetics was further examined through varying the rate of swelling as the solvent was uptaken into the films. This was achieved by setting different values of the stage temperature (between 15.9 and 20.9 °C, see Figure S6) during the initial solvent uptake component of the annealing, with a constant bubbler temperature and N_2_ flow as before. Figure 6 shows AFM images of the BCP film with the same *D*_0_ value of ∼166 nm annealed to approximately ∼5*D*_0_ (ϕ_s_ = 0.80) or ∼7*D*_0_ (ϕ_s_ = 0.86). The rate of solvent uptake was measured as the change in solvent concentration inside the BCP film over time, dϕ_s_/d*t*, and was calculated by fitting a linear regression model to the metered solvent uptake regime of the plots (example plots shown in Figure 6g), ignoring the initial solvent uptake regime between ϕ_s_ values of 0–0.25.^39^ Once the desired film thickness was reached, minor adjustments were made to the stage temperature in order to maintain a constant thickness value for ∼200 s, before rapid deswelling.

In the case of Figure 6a–c, where the films were swollen to a ϕ_s_ value of 0.8, the rate of swelling was varied from 1.2 × 10^–3^ to 6 × 10^–3^ s^–1^ (black square data points in Figure 6h). No significant structural change was observed as the value of dϕ_s_/d*t* was varied, and the BCP films remained in the region of the partially self-assembled state. A slight improvement in the calculated value of ξ is observed for films with ϕ_s_ = 0.8 in Figure 6h. Figure 6d–f shows improvement in ordering as the swelling rate decreased from 2.7 × 10^–3^ to 1.1 × 10^–3^ s^–1^ (red circle data points in Figure 6h), with the value of ξ increasing from 449 up to 724 nm.

A definitive explanation for this observation is difficult to ascertain, as less previous studies have investigated the influence of swelling rate on the ordering of BCP films. One possibility is that a slower swelling rate may result in less trapping of structural defects, thus resulting in larger ξ values. Nonetheless, a previous study has noted that the swelling rate did not noticeably influence their results for a smaller BCP system.^40^ Hence, we believe that future, more detailed kinetic studies are required to fully interpret these experimental results. It should be noted that the range of swelling rates examined for the ϕ_s_ = 0.86 sample set is smaller than for ϕ_s_ = 0.8. This is because it was found that our reflectometer model began to fail at swelling rates that exceeded approximately 3 × 10^–3^ s^–1^ for the films swollen to ϕ_s_ = 0.86. We suggest that this may be the result of increased macroscale film roughness caused by the rapid and non-uniform absorption of solvent into the film during a fast initial swelling regime, which is likely exacerbated at higher ϕ_s_ values. This established an experimental limit for the highest possible swelling rate shown in this work. It may be feasible to further accelerate the self-assembly process with a more uniform solvent distribution system in the chamber or different instrumentation to account for a large roughness factor during the in situ measurement of film thickness.

**Figure 7:**
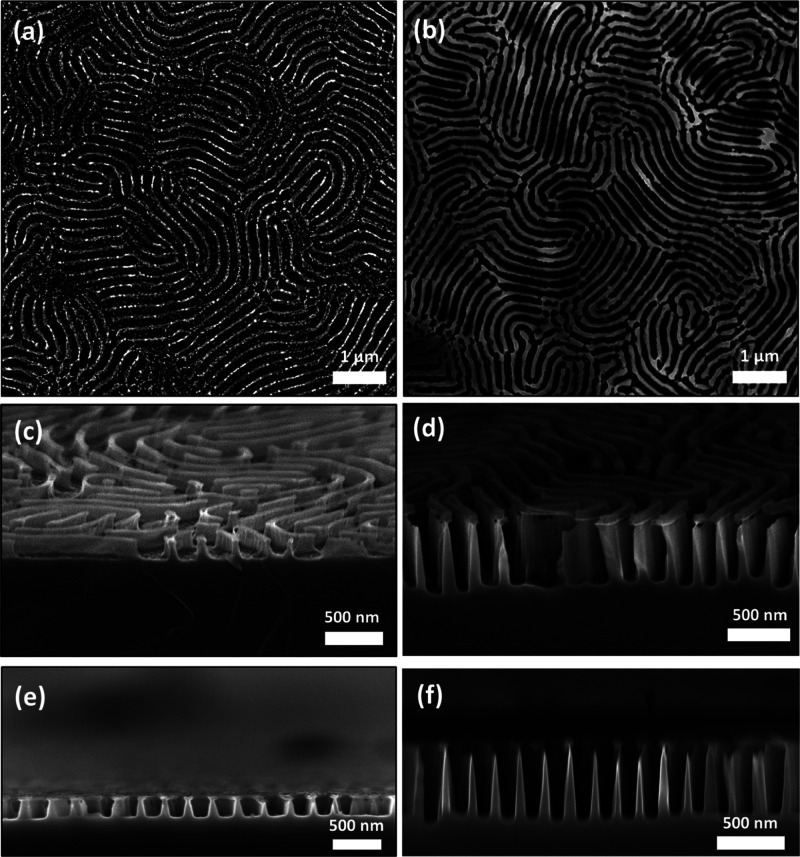
SEM images of Si nanowall after pattern transfer. Top-down SEM images of Si nanowall structures following an ICP etch of the iron oxide hard mask for (a) 1 and (b) 3 min etch, with tilted images (70°) shown in (c,d), respectively. (e,f) Cross-sectional SEM image of Si nanowalls for samples (a,b).

In order to demonstrate the pattern transfer capability of the self-assembled films, a liquid-phase metal salt infiltration process followed by UV/O_3_ treatment (to remove the polymer matrix) was performed to convert the BCP film into template for the formation of metal oxide nanostructures. This type of hard mask fabrication process has proven highly successful in previous work for manufacturing a variety of Si nanostructures.^12,60,61^ The metal oxide templates were then used as an etch mask, and the samples were etched for a range of times via an ICP-RIE plasma etching technique using CHF_3_ and SF_6_ process gases. In the case of samples with etch times of up to 1 min, an iron oxide hard mask was employed via infiltration of iron nitrate (Fe(NO_3_)_2_), while for etch times of up to 3 min, a nickel oxide mask (from a nickel nitrate precursor) was instead used. This is because it was found that the nickel oxide mask was more durable over the longer etch times and resulted in less structural degradation and higher etch contrast. Figure 7a–f shows the SEM images of the resulting Si nanowall structures after 1 and 3 min, demonstrating a high degree of homogeneity over the sample surfaces. Figure 7e,f shows the cross-sectional SEM images of the Si nanowall features, again showing uniform nanowall structures with feature spacings of ∼190 nm and heights of ∼145 and ∼493 nm. The aspect ratio of the features shown in the 3 min etched sample (Figure 7f) was measured to be approximately 7.5. The successful pattern transfer of the BCP film and generation of vertical sidewalls furthermore confirm the efficacy of the optimized SVA process. A summary table of the variation of feature heights with etch time is displayed in Figure S13, along with high-resolution cross-sectional SEM images of the nanowall structures.

## Conclusions

In order for BCP lithography to become a viable candidate for the fabrication of large period (>100 nm) nanostructures, it is critical that the self-assembly of UHMW systems be enhanced for maximal structural control and minimal annealing time. We studied the swelling kinetics of an UHMW BCP system through the use of a temperature-controlled solvent annealing rig, allowing for precise control over the pattern structure, ordering, over a very short period of annealing time. We show that at very high levels of solvent uptake (ϕ_s_ ≥ 0.8), where the polymer concentration is close to the experimentally estimated ODT point, it is possible for an UHMW BCP system to undergo rapid phase separation into lamellar domains of 190 nm spacing on timescales of 10 min. The closer the value of ϕ_s_ becomes to the observed ϕ_s,ODT_ value, the higher the lateral ordering of the film. The thickness of the dry BCP film was found to positively correlate with both the minimum ϕ_s_ value required to initiate self-assembly, along with the observed ODT value of ϕ_s_. Furthermore, when *D*_0_ was modulated in conjunction with ϕ_s_, the domain morphology could be induced to obtain the kinetically unstable HPL phase at a thickness close to half that of the domain period.^12^ The amount of time a polymer film is held at a particular value of ϕ_S_ is shown not to appreciably impact the ordering or domain structure. The rate of solvent uptake, on the other hand, exhibits a moderate influence on the domain ordering for ϕ_s_ values that are sufficiently high to induce phase separation, with slower swelling rates resulting in improved lateral ordering. It should be noted that the optimization of lateral ordering was somewhat limited by the temperature sensitivity of our annealing setup; we anticipate that future studies may achieve even higher degrees of ordering through controllably approaching the ODT point with greater accuracy. The domain structures obtained from these self-assembled BCP films were easily utilized for pattern transfer through metal salt inclusion, with the formation of metal oxide hard masks that were etched to create uniform and regular arrays of high aspect ratio Si nanowall features.

In conclusion, the results of this study demonstrate that a considerable reduction in the annealing time of UHMW BCP systems is possible using a carefully regulated SVA-based approach, improving upon previous work that typically required high-temperature annealing and/or timescales on the order of hours or days.^20,28−30^ The speed and reliability of this technique represents a major step toward a cost-effective and scalable strategy for the fabrication of optical nanostructures such as 2D photonic crystal structures^62,63^ using BCP lithography, with feature spacings on the order of visible light.

## Experimental Section

### Materials and Sample Preparation

Polystyrene-*block*-poly(2-vinylpyridine) (*M*_n_: 440-*b*-353 kg/mol, PDI: 1.19) was purchased from Polymer Source Inc. and used without further purification. Anhydrous toluene, THF, and ethanol were purchased from Sigma-Aldrich and used without further purification. Varying amounts of PS-*b*-P2VP were dissolved in a 4:1 (volume fraction) mixture of toluene and THF to make polymer solutions between 0.5 and 3% (w/w), which were left stirring overnight to ensure complete dissolution. For SVA, chloroform and THF (both HPLC, 99.9%, Sigma-Aldrich) were used. 2 × 2 cm pieces of Si⟨100⟩ wafers with a native oxide layer were cleaned by ultrasonication in acetone for 20 min, followed by drying under N_2_ gas. BCP solutions were then spin-coated onto the clean Si substrates for 30 s at 4500 rpm.

### Annealing Rig

The SVA rig utilized in this study is an upgraded version of the setup described by Lundy et al.^38^ The stainless steel annealing chamber has an internal volume of 1.94 L and possesses an access door with a quartz glass viewport located on the top of chamber (see Figure 1). The inlet and outlet valves for solvent/N_2_ vapor flow are located on the left- and right-hand side of the chamber, respectively. To generate solvent vapor, nitrogen gas was passed through a flow meter and into a bubbler chamber containing the THF–chloroform mixture. In order to maintain a constant vapor pressure inside the SVA chamber over the entire annealing process, it was essential to mitigate any temperature decrease of the solvent mixture over time due to evaporative cooling. This was achieved by attaching a flexible heat pad to the solvent bubbler. The heat pad was connected to a PID controller, which maintained a constant solvent temperature of 21.0 ± 0.15 °C using feedback from a resistance temperature detector (RTD) probe located inside the bubbler chamber as shown in Figure 1. The flow rate of the N_2_ gas was held at ∼400 sccm during annealing. The solvent chamber could be rapidly quenched to preserve the phase-separated BCP morphology using a N_2_ purge line. A copper stage is located inside the chamber upon which the samples were placed during annealing, allowing samples of up to 4″ to be processed. An RTD embedded inside the copper stage allowed the stage temperature to be measured and provided feedback for a PID controller to control the stage temperature (±0.15 °C). In order to monitor the film thickness in situ during SVA, a Filmetrics F3-CS reflectometer with a UV–vis light source (380–1050 nm) was mounted on top of the quartz viewport. To account for any variation in the light source intensity, the reflectometer was calibrated using a Si reflectance standard prior to each sample run. The experimental reflectance data was measured over a wavelength range of 420–1050 nm and used a three-layer model consisting of the Si substrate, the PS-*b*-P2VP BCP layer, and air. Refractive index models for both the dry BCP films and the swollen films were estimated using the Lorenz–Lorentz rule of mixing, which utilized the refractive indices of the pure polymer and solvent components (PS, *n* = 1.586, P2VP: *n* = 1.527, THF: *n* = 1.407, and chloroform: *n* = 1.440) to obtain a refractive index range of 1.558 (for the dry BCP film) to 1.457 as the concentration of the THF/chloroform solvent mixture increased up to a ϕ_s_ value of 0.87 (see Section S2). The time interval used for each data point shown was 2 s, with an integration time of ∼250 ms.

### SVA Process

The BCP samples were placed on the heated copper stage directly under the reflectometer beam, which was baselined prior to each sample measurement as mentioned above. The initial stage temperature, which ranged between 15.9 and 20.9 °C depending on the experiment was set prior to initiating the annealing process. Once the temperature had stabilized, solvent vapor was introduced at a rate of 400 sccm during which the film thickness was recorded in real time by the reflectometer (see Section S2 for fitting details). Once the sample approached the desired swollen thickness, the temperature of the stage was gradually increased in increments of 0.1 °C using the PID controller to slow the swelling rate to zero. The film was then held at this swollen thickness value for the required annealing time. During the hold time, the stage temperature was periodically adjusted in increments of 0.1 °C to avoid any large fluctuations in the film thickness. As soon as the required hold time was reached, the temperature of the stage was instantaneously increased to 30 °C and the chamber was purged with nitrogen, which ensured a rapid deswelling regime (of approximately 5–10 s depending on the swollen thickness) and that all the remaining solvent inside the film was purged.

### Oxide Nanostructure Formation and Pattern Transfer

The formation of the metal oxide structure from the BCP template follows a similar process described in previous work. The samples were first immersed in ethanol for 20 min to facilitate surface reconstruction and subsequently left to dry at room temperature. Iron(III) nitrate nonahydrate (Fe_2_(NO_3_)_3_·9H_2_O) was dissolved in ethanol at a concentration of 0.5% w/w, and nickel(II) nitrate hexahydrate (Ni(NO_3_)_2_·6H_2_O) was dissolved in ethanol at a concentration of 0.6 w/w %. The salt solution was stirred for 1 h to ensure complete dissolution and was then spin-coated onto the ethanol-reconstructed BCP samples at a speed of 3200 rpm for 30 s. A UV/O_3_ treatment (PSD Pro Series Digital UV Ozone System; Novascan Technologies, Inc.) was then performed on the samples for 3 h to completely remove the polymer template and oxidize the metal precursor. The patterns were etched into the substrate using an OIPT Plasma lab System100 ICP180 etch tool utilizing a gas mixture of SF_6_ (15 sccm) and CHF_3_ (80 sccm), an ICP power of 1200 W and RIE power of 20 W, and a chamber pressure of 20 mTorr.

### Sample Characterization

AFM was performed in non-contact mode [Park Systems, XE-7 under ambient conditions using silicon cantilevers (PPP-NCHR model)] with a force constant of 42 N/m. SEM images were taken using a Carl Zeiss Ultra plus using an InLens detector with an accelerating voltage of 5 kV and a working distance of 4.5 mm. For cross-sectional SEM images, the substrate was cleaved into two and placed on a sample holder that was angled perpendicular to the electron beam. The stage was then tilted to 10–20° depending on the image. GISAXS was performed at the Soft Matter Analytical Laboratory (SMALL), Department of Chemistry, University of Sheffield, using a Xeuss 2.0 (Xenocs) system with 9.243 keV X-rays from a liquid Ga MetalJet source (Excillum) with an incident beam angle of 0.16°. The sample to detector distance was 6.404 m, flight tubes were held under vacuum to remove air scatter, and the data was processed using Foxtrot Soleil and the GIXSGUI MATLAB Toolbox.^64^

### Image Analysis

Orientational mapping was performed on SEM images using the OrientationJ plugin for ImageJ. The correlation length, an indicator of orientational ordering in the pattern, was estimated using software described by Murphy et al.,^52^ where the correlation function is obtained from a set of orientation angles that are calculated using a skeletonization process on the BCP domain structures of each image. 10 × 10 μm AFM images were used to determine the correlation length values shown in this report (example in Figure S12). To briefly describe the process, the contrast between the two BCP domains (in this case the PS and P2VP domains) is first enhanced through smoothening (to reduce random noise) and then converted into a binary image by thresholding (PS represented as black, P2VP white). The line features identified from the binary images are then skeletonized into single-pixel width, and the orientational angle ϕ(*r*) of each pixel along the skeletonized line features is determined using a rolling average of the tangent along each line. The correlation function *C*(*r* – *r*′) can then be calculated from the set of orientation angles ϕ(*r*) for each point analyzed in the image as follows: *C*(*r* – *r*′) = cos[2{ϕ(*r*) – ϕ(*r*′)}]. The correlation length ξ is then related to the correlation function through an exponential fit: *C*(*r* – *r*′) = e^–*r*/ξ^. All error bars in this work represent two standard deviations (2σ) from the mean value unless stated otherwise.

## Supporting Information Available

Estimation of the partial pressures of THF/chloroform mixtures inside the SVA chamber; calculation of refractive index of swollen film; additional swelling plots; additional AFM and SEM data; optical micrographs of BCP films post SVA; correlation length example; and feature height variation with etch time (PDF)
