# Supplementary material for: Optimization and Control of Large Block Copolymer Self-Assembly via Precision Solvent Vapor Annealing
Source: Macromolecules. 2021 Jan 22;54(3):1203–15. doi: 10.1021/acs.macromol.0c02543 (PMC8280752; doi:10.1021/acs.macromol.0c02543)
Supplement: Supplementary file 1 — ma0c02543_si_001.pdf [file ma0c02543_si_001.pdf]

Supporting Information

Optimization and Control of Large Block Copolymer Self-Assembly via Precision Solvent Vapor Annealing

Andrew Selkirk,[a,b] Nadezda Prochukhan,[a,b] Ross Lundy,[a,b] Cian Cummins,[c] Riley Gatensby,[a,b] Rachel Kilbride,[d] Andrew Parnell,[d] Jhonattan Baez Vasquez,[a,b], Michael Morris,[a,b] Parvaneh Mokarian-Tabari [a,b*]*

[a] Advanced Material and BioEngineering Research Centre (AMBER), Trinity College Dublin, The University of Dublin, Ireland.

[b] School of Chemistry, Trinity College Dublin, The University of Dublin, Dublin 2, Ireland.

[c] CNRS, Bordeaux INP, LCPO, UMR 5629 and CNRS, Centre de Recherche Paul Pascal, UMR 5031, Univ. Bordeaux, F-33600 Pessac, France.

[d] Department of Physics and Astronomy, University of Sheffield, Sheffield, S3 7RH, UK.

Section S1: Estimation of the partial pressures of THF:chloroform mixtures inside the SVA chamber

In order to fully optimize the SVA process of our UHMW system, it was essential to deduce the optimal choice of solvent to obtain phase separation into lamellar domains. The primary solvents investigated were chloroform and THF, due to their relative neutrality towards both PS and P2VP domains. In an ideal solvent mixture, the vapour pressures of each solvent would exactly match the concentration of the solvent in the liquid mixture as per Raoult's law. Chloroform/THF mixtures are non-ideal, however, and therefore the activity coefficient (γ) of a solvent in a binary mixture must be considered in order to calculate the partial pressure:

$$p_i = \gamma_i \, x_i \, p_{i,sat} \tag{1}$$

Where x_i is the molar fraction of solvent i in the liquid phase, and $p_{i,sat}$ is the saturated vapour pressure of the pure solvent i. $p_{i,sat}$ can be estimated from the Antoine equation:

$$\log(p_{i,sat}) = A_i - \frac{B_i}{C_i + T} \tag{2}$$

Where A_i, B_i and C_i are the component-specific constants, and T is the temperature. The values of these constants for chloroform at 21°C are $A = 6.995, B = 1202.29, C = 226.25$, and for chloroform are $A = 6.955, B = 1170.97, C = 226.232$ [2, 3]. The molar ratio of both solvents in the gas form (y) can be calculated using the following expression:

$$y_i = \frac{p_i}{p_{total}} \tag{3}$$

Where p_{total} is the total pressure of the solvent mixture. The behaviour of chloroform and THF in a binary mixture has been studied in past work. At temperatures of between 20-30°C it has been found that the mixture displays a negative deviation from ideality, meaning that the total pressure of the mixture passes through a minimum value as the composition of the mixture is changed [4, 5].

To analyse the effect of x_i on the phase separation, the values of γ_i for each molar ratio of solvent examined were required. Previous experimental work on chloroform/THF binary mixtures determined that an athermal model based on Flory-Huggins theory can accurately predict the values of γ_i obtained from experimental results.[1, 6] The values of γ_i from this model for both chloroform and THF were used to determine the partial pressures of both solvents in the vapour phase during annealing (see **Figure S1**). Various ratios of both solvents were added to the solvent bubbler and used to swell the block copolymer films to a constant ϕ_S value of ~0.83. All other parameters, including swelling rate, initial stage temperature, and swelling time, were held constant.

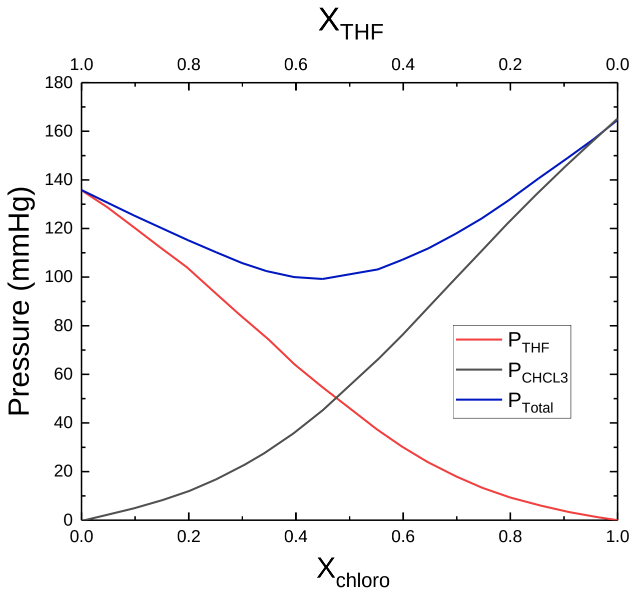

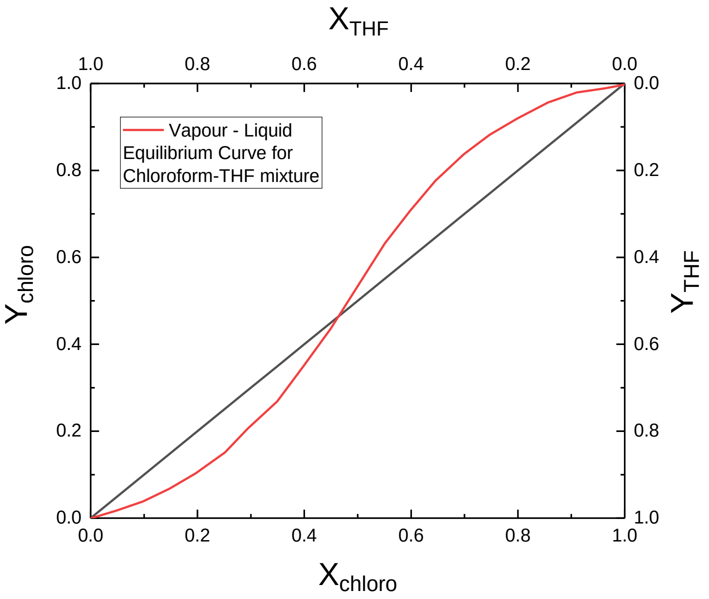

Figure S1: (a) Graph of the vapour pressures of chloroform (P_{CHCl_3}), THF (P_{THF}), and the total equilibrium vapour pressure P_{total} vs. the concentration of chloroform in solution (x_{CHCl_3}) at 294.15K. Data for the values of the activity coefficients γ_i were obtained from *Dohnal* et al.[1]
(b) Vapour liquid equilibrium curve of the THF-chloroform mixture.

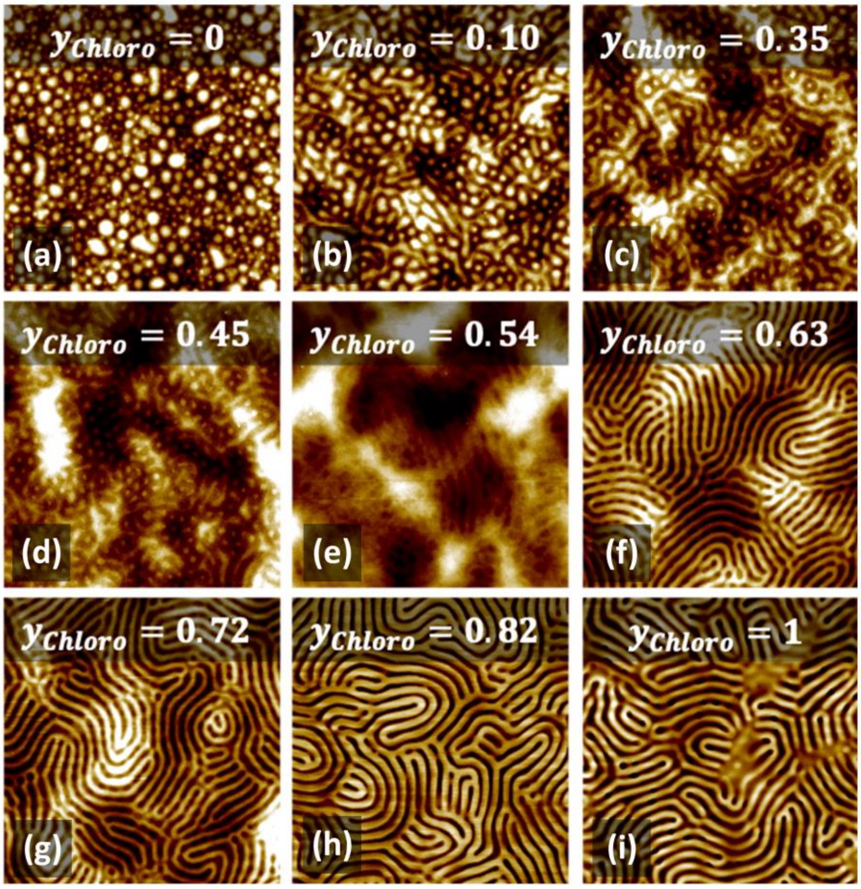

Figure S2: AFM images (5 × 5 μm) of PS-b-P2VP films annealed in the rig for 200s to a ϕ_s value of ~0.83 using a variety of chloroform/THF ratios. The calculated molar fraction of chloroform y_{CHCl_3} in the vapour phase is shown inset for each image.

Figure S2 shows AFM images of the BCP films after 200s of swelling to a ϕ_s value of ~0.83, with bubbler temperature of 21C. In the case of pure THF, the film appeared to remain in the original 'as cast' micellar form after SVA. A similar structure is observed when $y_{CHCl_3} = 0.1$, which progresses to a partially phase-separated structure at $y_{CHCl_3} = 0.54$, and eventually to the expected lamellar structure as the chloroform becomes the majority component.

Although the lamellar form was observed using pure chloroform, the solvent mixture that gives $y_{CHCl_3} = 0.82$ was utilised for the kinetic studies outlined in the main text. This is because despite multiple attempts of swelling to high ϕ_s values using pure chloroform, the resulting lamellar structure was observed to contain nano-scale regions of non-uniformity where the ordering was lost, as can be seen in (g). We suggest that this may be the result of increased difficulty in maintaining a constant swelling profile across the film due to the ~20% higher overall equilibrium vapour pressure of pure chloroform vs. the solvent mixture with $y_{CHCl_3} = 0.82$ at 21°C (calculated from figure 2 values). This is an interesting observation in itself, as it suggests that negative azeotropic solvent mixtures may be better suited for maintaining thickness control at high degrees of swelling where the swollen film thickness becomes increasingly sensitive to temperature changes. A more in-depth examination of the effect of solvent mixtures on film swelling kinetics will be the subject of a future study.

Section S2: Calculation of Refractive Index of Swollen Film

The refractive index values of the solvent mixture, pure BCP film, and swollen BCP film were estimated from their known pure component values (see table 1) using the Lorenz-Lorentz rule of mixing[7].

$$\frac{n_{12}^2 - 1}{n_{12}^2 + 2} = \phi_1 \frac{n_1^2 - 1}{n_1^2 + 2} + \phi_2 \frac{n_2^2 - 1}{n_2^2 + 2} \tag{4}$$

Where ϕ_1, ϕ_2 and n_1, n_2 are the volume fractions and refractive indices of pure components 1 and 2, and n_{12} is the refractive index of the resulting mixture.

Component	Refractive index value:
Tetrahydrofuran[8]	1.405 ± 0.001
Chloroform[9]	1.446 ± 0.001
Polystyrene[10]	1.587 ± 0.001
2-vinylpyridine[11]	1.550 ± 0.001

For the molecular weight of PS-*b*-P2VP used in this work, a refractive index value of 1.570 was obtained using the respective volume fractions of each block. For the chloroform/THF mixture, an approximation was made that the ratio of each solvent absorbed into the film during swelling is equal to the solvent ratio in the vapour phase. This yields a value of 1.438 using eq. (4) for $y_{CHCl_3} = 0.82$. For the swollen film, refractive index values of between 1.503 and 1.453 were obtained for ϕ_s values between 0.5 to 0.88 respectively via incorporating the refractive indices of the both the solvent mixture and block copolymer film.

During a swelling experiment, the refractive index was initially set at a precalculated profile corresponding to the dry film (n=1.438, **figure S3a**). Once swelling was induced, the precalculated refractive index profile was switched (within the Filmetrics software) to the corresponding index profile of the desired solvent concentration of the swollen film for that experiment (examples in **figure S3b, c**). Upon initiation of deswelling, the refractive index profile was immediately reverted back to that of the dry film. This ensured that our reflectometer model aligned with the measured BCP film during the experiment. We intend to automate such refractive index changes in our future studies via a feedback loop system in order to further enhance the scalability and precision of the technique

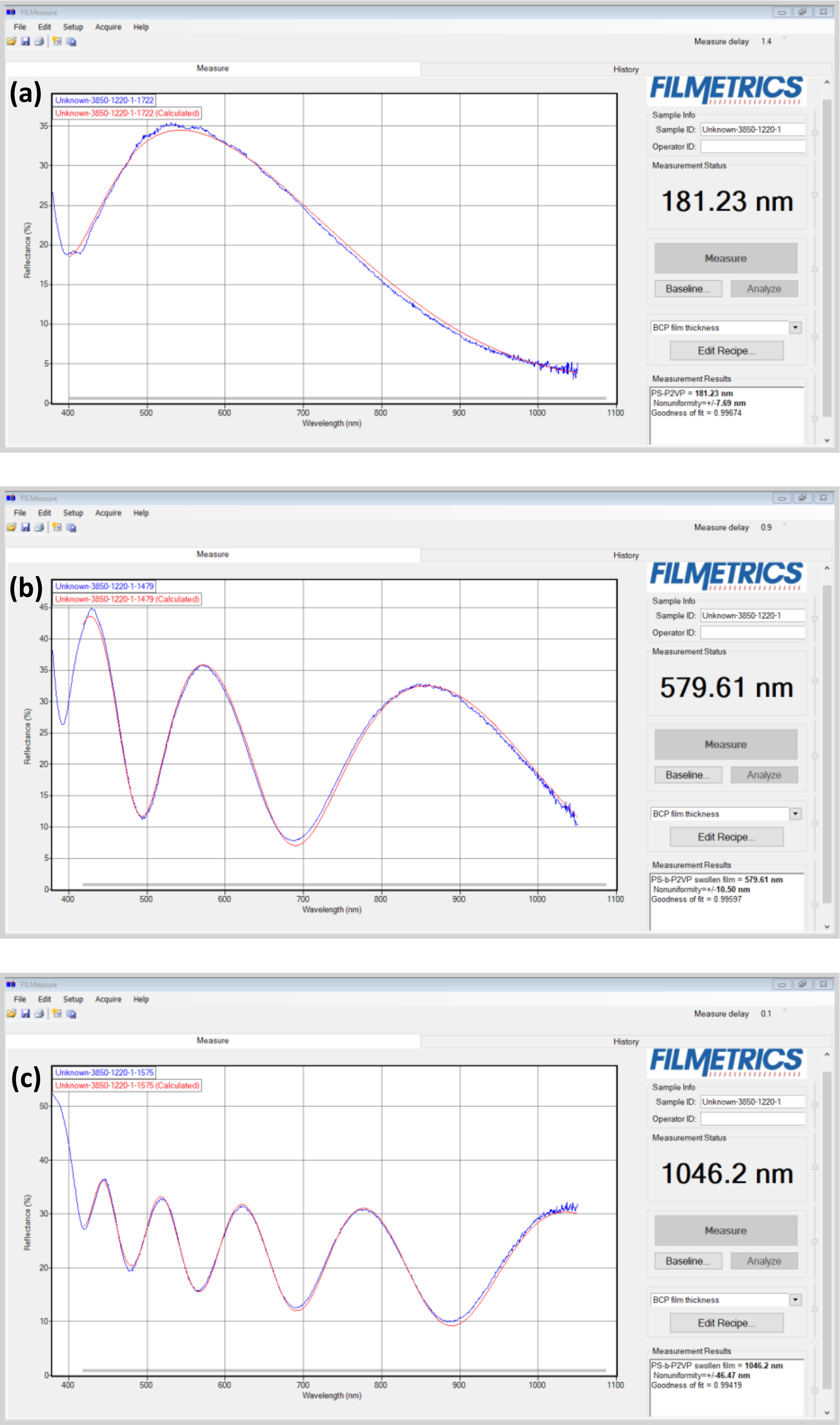

Figure S3: Spectral reflectance of the unswollen neat BCP film (a), the film swollen to a ϕ_s value of ~0.69 (b), and swollen to a ϕ_s value of ~0.83 (c).

Section S3: Additional Swelling Plots

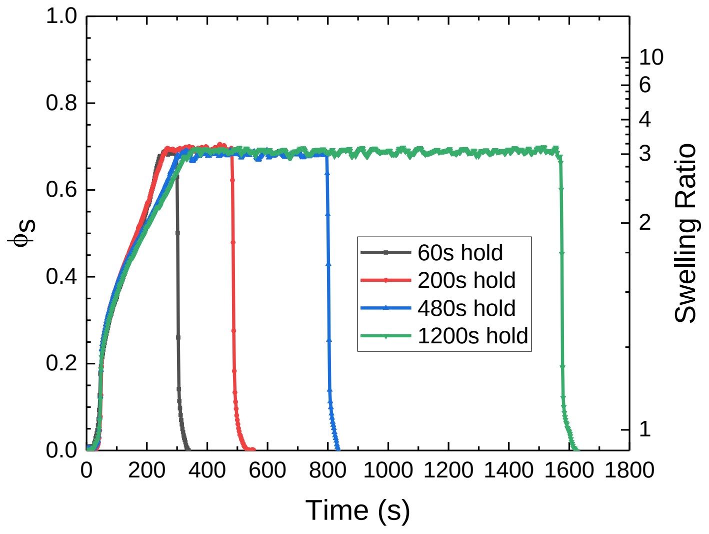

Figure S4: Swelling plot of the films shown in figure 5 (a) to (e) of the main text.

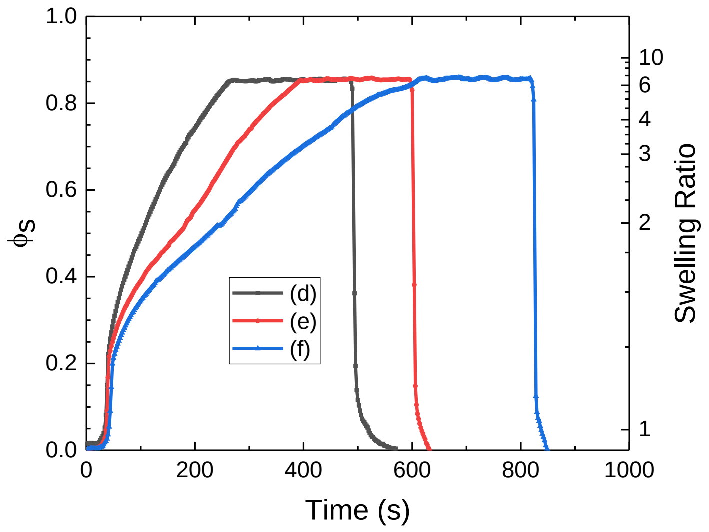

Figure S5: Swelling plot of the films shown in figure 6 (d) to (f) of the main text.

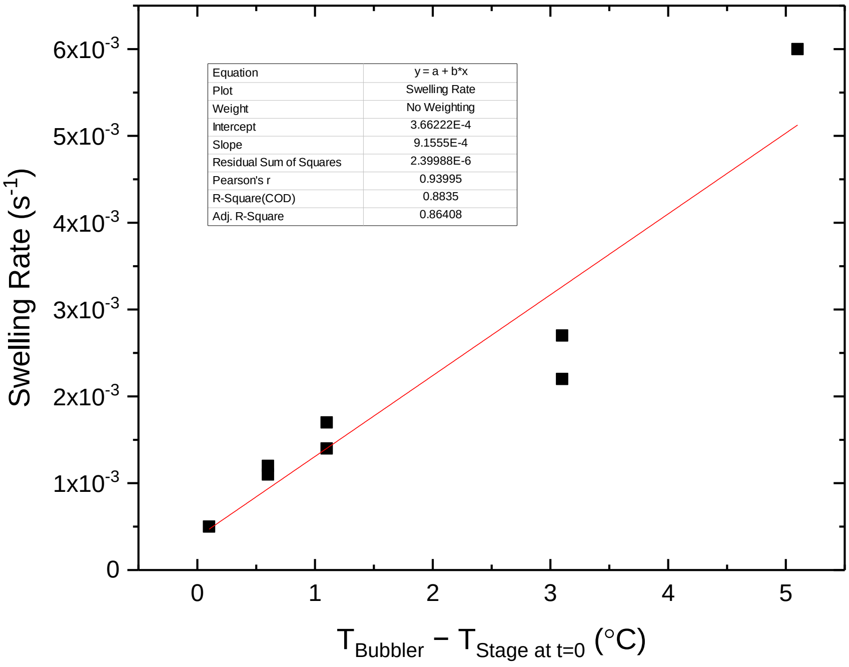

Figure S6: Plot of the calculated swelling rate vs. the difference between the initial stage temperature (varied between $15.9°C$ to $20.9°C$) and bubbler temperature (21°C for all samples).

Section S4: Additional AFM, SEM data:

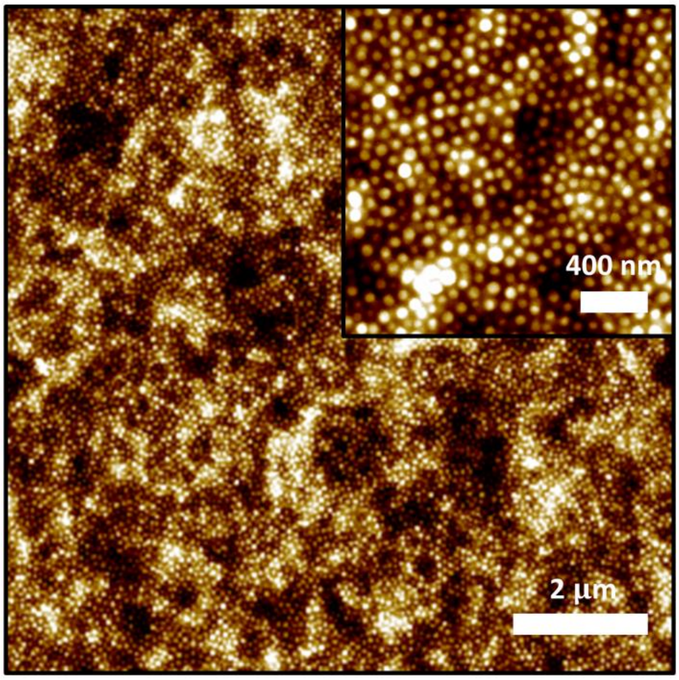

Figure S7: AFM image of the as cast PS-*b*-P2VP film.

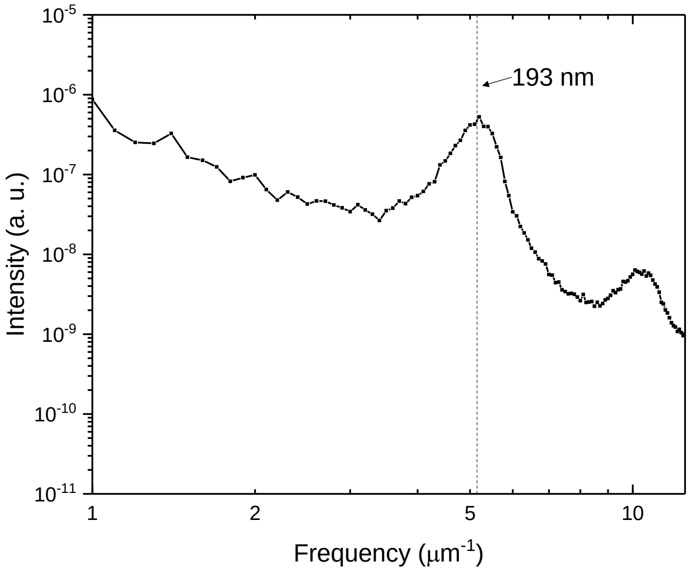

Fig. S8: 2-dimensional **p**ower spectral density (PSD) plot of an AFM image of the BCP film shown in sample 2 (n). The peak corresponding to the feature spacing of 193 nm is marked, and was determined using a Gaussian fit.

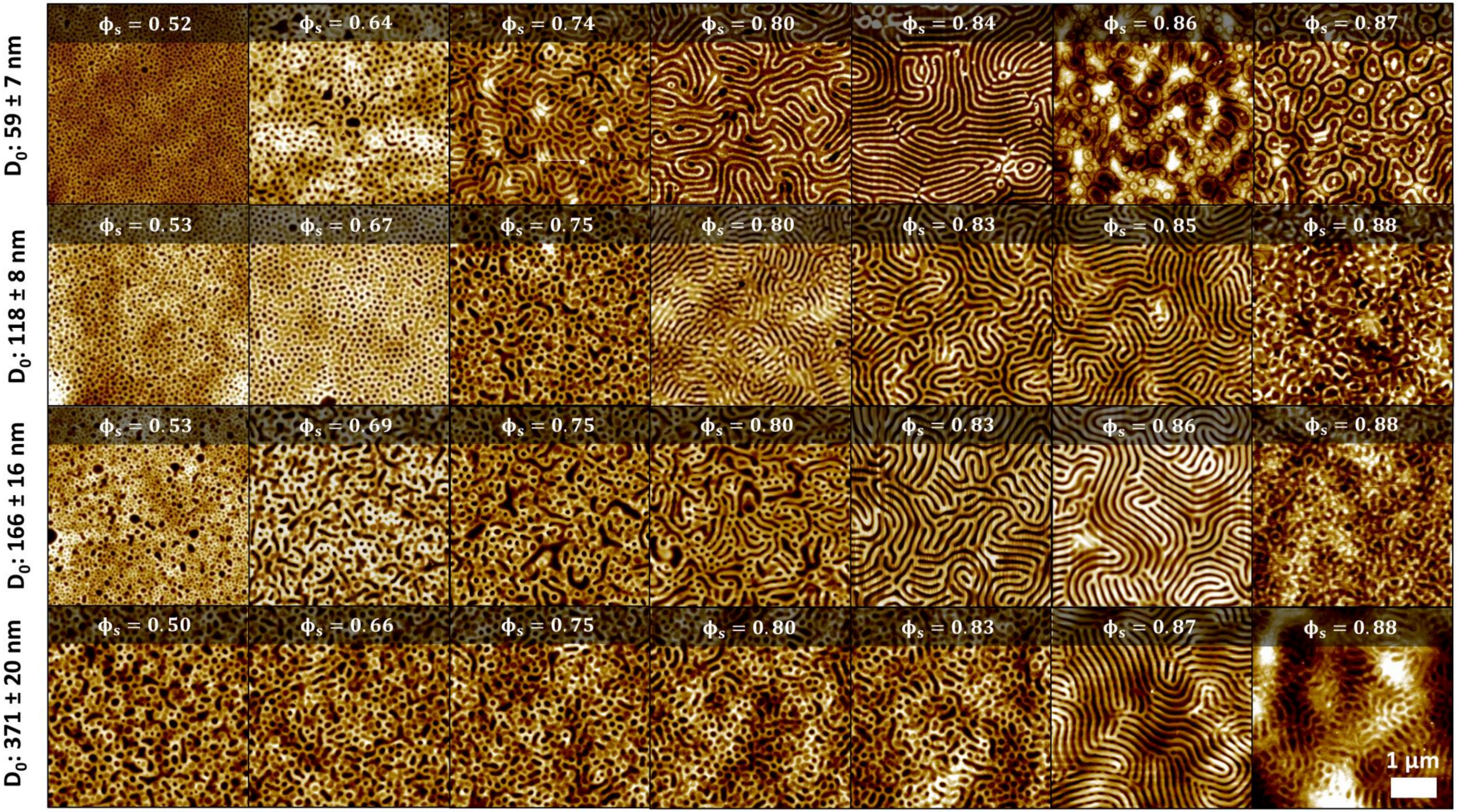

Figure S9: AFM images of the PS-*b*-P2VP films as a function of the solvent concentration in swollen film ϕ_s, and initial film thickness D_0. These images were used to construct the orientation diagram shown in fig. 2 in the main text.

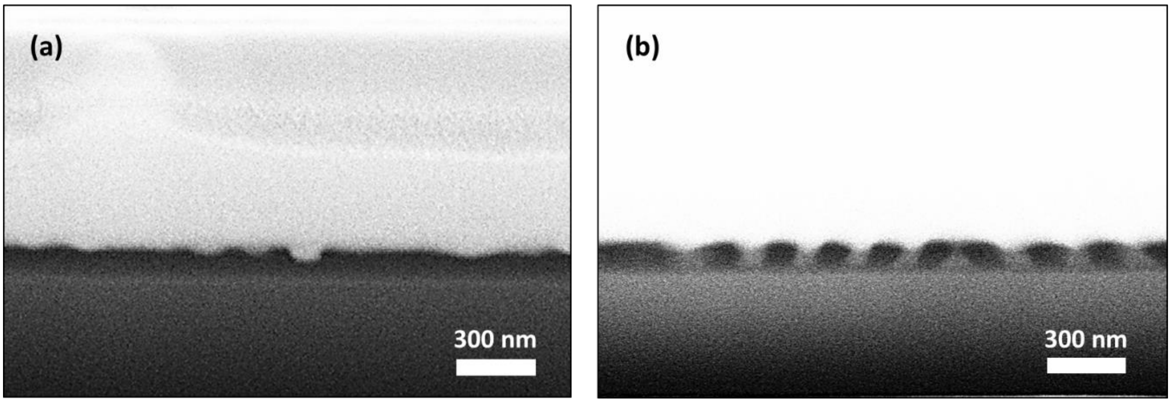

Figure S10: FIB/SEM images of 166 nm thick PS-*b*-P2VP films after a total annealing time of 10 mins at a ϕ_s value of (a) 0.67 and (b) 0.86.

Section S5: Optical Micrographs of BCP films Post SVA

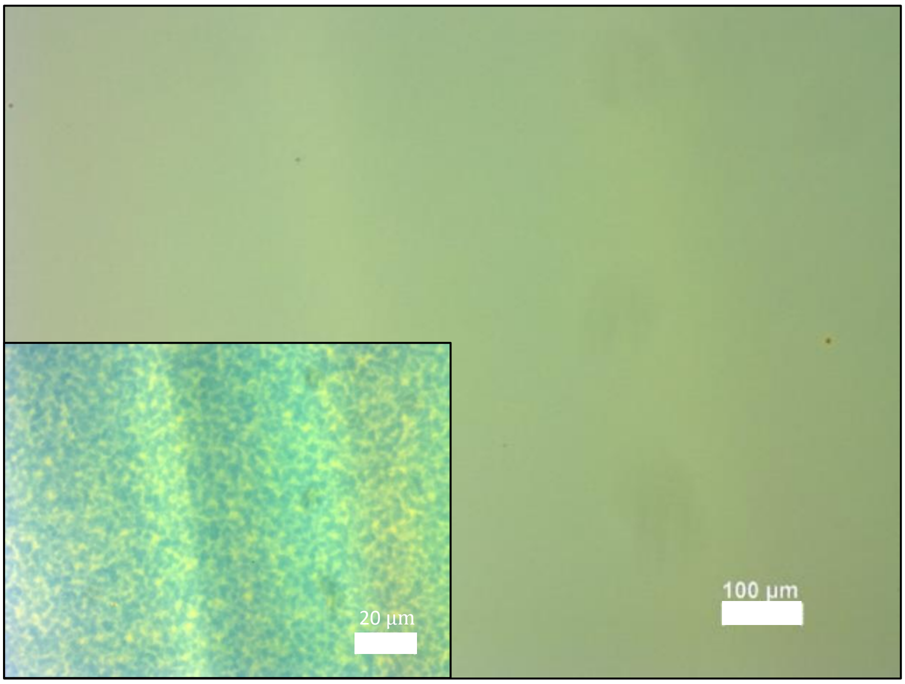

Figure S11: Optical microscopy images of the PS-*b*-P2VP film after a total annealing time of 10 mins at a ϕ_s value of 0.86 (giving lamellar orientation). Image is taken after SVA with no surface reconstruction.

Section S6: Correlation Length example:

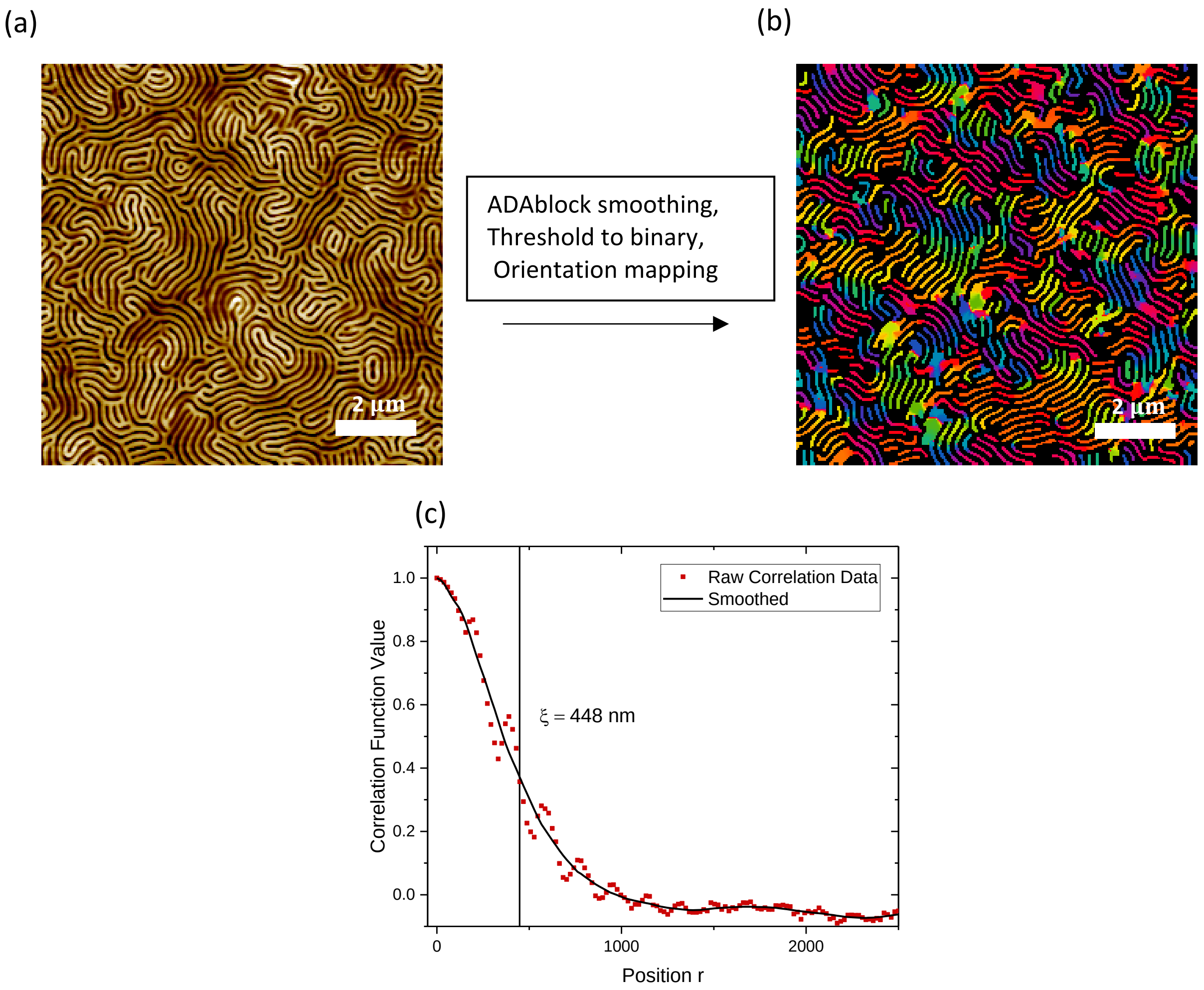

Figure S12: (a) Example $10 \times 10\ \mu m$ AFM image (from film shown in figure 6 (d) of main text). (b) Generated orientational map of skeletonized AFM image as generated from the ADAblock application.[12] (c) Calculation of correlation function from AFM image. Raw data is shown in red with smoothed data in black. Correlation length is marked at the r value where the correlation function is equal to $1/e$

S7: Feature height variation with etch time:

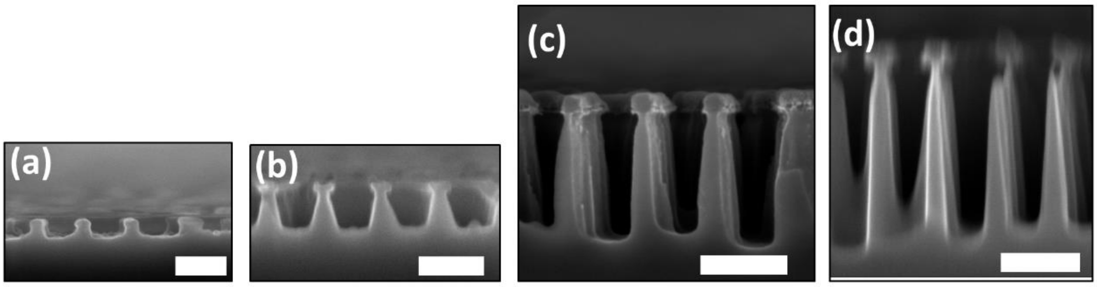

Etch time (s)	30	60	120	180
Height (nm)	78	145	373	493

Figure S13: High resolution cross-sectional SEM images of Si nanowalls, after (a) 30 seconds, (b) 60 seconds, (c) 120 seconds, and (c) 180 seconds of etch time. The table shows the variation of feature heights with etching time. All scale bars are 200 nm.

Bibliography:

1. Dohnal, V.; Costas, M., Thermodynamics of complex formation in chloroform-oxygenated solvent mixtures. *Journal of Solution Chemistry* **1996,** *25* (7), 635-656.
2. Smallwood, I., *Handbook of Organic Solvent Properties*. Elsevier: Oxford, 1996.
3. Stull, D. R., Vapor Pressure of Pure Substances. Organic and Inorganic Compounds. *Industrial & Engineering Chemistry* **1947,** *39* (4), 517-540.
4. Oswal S.L., D., D. D, Excess Free Energies & Excess Volumes for Chloroform + Tetrahydrofuran Systems. *Indian Journal of Chemistry -Section A* **1978,** *16*, 798-800.
5. Byer, S. M.; Gibbs, R. E.; Van Ness, H. C., Vapor-liquid equilibrium: Part II. Correlations from P-x data for 15 systems. *AIChE Journal* **1973,** *19* (2), 245-251.
6. Dohnal, V.; Fenclová, D.; Bureš, M.; Costas, M., Thermodynamic study of complex formation by hydrogen bonding in halogenoalkane–oxygenated solvent mixtures: halothane with acetone, methyl acetate, tetrahydrofuran and methyl tert-butyl ether. *Journal of the Chemical Society, Faraday Transactions* **1993,** *89* (7), 1025-1033.
7. Heller, W., Remarks on Refractive Index Mixture Rules. *The Journal of Physical Chemistry* **1965,** *69* (4), 1123-1129.
8. Lide, D. R., *CRC Handbook of Chemistry and Physics, 88th Edition.* CRC Press Inc.: Boca Raton, Florida, 2008.
9. Haynes, W. M., *CRC Handbook of Chemistry and Physics, 95th Edition*. CRC Press Inc.: Hoboken, 2014.
10. Sultanova, N. G.; Kasarova, S. N.; Nikolov, I. D., Dispersion Properties of Optical Polymers. *ACTA PHYSICA POLONICA A* **2009,** *116*, 585-587.
11. Weast, R. C., *CRC Handbook of Chemistry and Physics, 60th Edition.* CRC Press Inc.: Boca Raton, Florida, 1979.
12. Murphy, J. N.; Harris, K. D.; Buriak, J. M., Automated Defect and Correlation Length Analysis of Block Copolymer Thin Film Nanopatterns. *PLOS ONE* **2015,** *10* (7), e0133088.
